# Braess’ Paradox in Enzyme Kinetics: Asymmetry from Population Balance without Direct Cooperativity

**DOI:** 10.1021/acs.jctc.5c01269

**Published:** 2026-02-04

**Authors:** Malte Schäffner, Colin A. Smith, Robert Tampé, Helmut Grubmüller

**Affiliations:** † Theoretical and Computational Biophysics Department, 28282 Max Planck Institute for Multidisciplinary Sciences, Am Fassberg 11, Göttingen 37077, Germany; ‡ Institute of Biochemistry, Biocenter, Goethe University Frankfurt, Max-von-Laue-Str. 9, Frankfurt Am Main 60438, Germany

## Abstract

The ATPase ABCE1, a member of the ubiquitous ATP-Binding Cassette protein superfamily, is essential in eukaryotic and archaeal ribosome recycling. It comprises a pair of homologous nucleotide-binding domains (NBDs), each containing a consensus nucleotide-binding site (NBS), where ATP hydrolysis takes place. Each of these sites can be in either an open or closed conformation. Despite the near symmetry of the two NBDs, and quite unexpectedly, their hydrolysis kinetics are highly asymmetric. While substitution of the catalytic glutamate (E238Q) in NBSI reduced the overall turnover rate of the ATPase by a factor of 2, as one might expect, the corresponding substitution in NBSII (E485Q) shows a so far unexplained 10-fold increase. To address this issue, we used Markov models to study how such a drastic asymmetry can arise. Specifically, we asked whether this observation can be explained without previously proposed direct allosteric interactions, such as electrostatic interactions, between the two NBSs. Indeed, using a Bayesian approach, we found Markov models that quantitatively predict the experimentally observed kinetics, as well as additional steady-state ATP occupancy data, both without such direct allosteric interaction. In particular, our results show that the observed remarkable asymmetry is fully explained by the structure-induced property that opening and closing always involves both NBSs. These models can explain the unexpected fast kinetics of the mutant of NBSII in terms of a drastic population shift due to the mutation, which circumvents a kinetic trap state that slows wild-type kinetics. Our Bayesian Markov approach may help to quantitatively explain similar nonintuitive Braess-type kinetics also in other enzymes where chemical/conformation coupling is essential.

## Graphical abstract

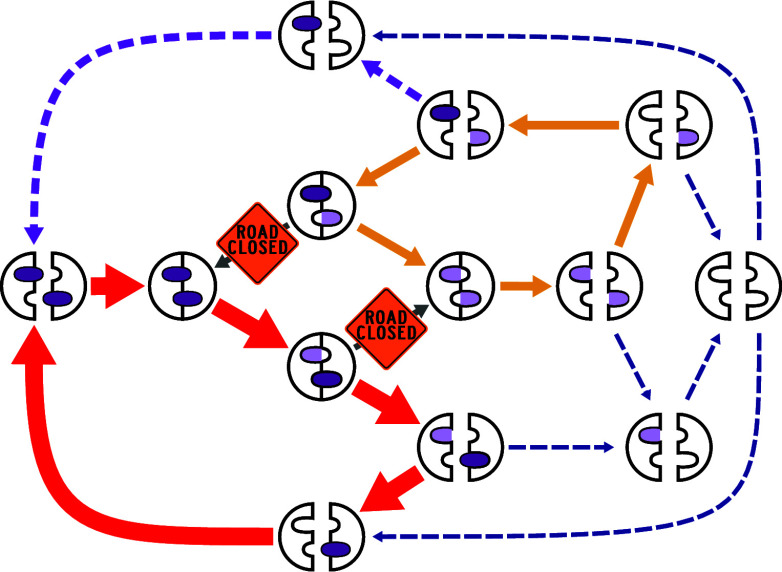

## 1 Introduction

The protein ATP-binding cassette (ABC) subfamily E member 1 (ABCE1)^1,2^ is a member of the ABC transporter superfamily. In ABC transporters, ABCE1 homologues^3^ serve as chemo-mechanic energy converters, which drive conformational changes of transmembrane domains, which in turn affect transport of the respective substrate.^4^ In this sense, the soluble protein ABCE1, which is void of transmembrane parts, is a prototype of the “molecular motor” of this large and crucial membrane protein family. In fact, based on the very high sequence conservation, being one of the most conserved proteins in evolution, ABCE1 might even be a prototype for a very ancient molecular motor. As an exception within this protein superfamily, ABCE1, in coordination with other translation factors, drives separation of the large and small ribosomal subunit via a drastic displacement of an N-terminal Fe_4_S_4_ cluster domain,^5–8^ which also involves chemo-mechanical energy conversion.

ACBE1 is mainly composed of two sequentially and structurally highly similar nucleotide-binding domains (NBDs).^7–14^ Figure 1A shows the structure of ABCE1, highlighting seven highly conserved sequence motifs present in each of the two NBDs, which are required for ATP-binding and hydrolysis, namely, the A-loop (Y-loop), Walker-A (P-loop), Q-loop, His-switch (H-loop), Walker-B, D-loop, and the ABC-signature motif (C-loop).

**Figure 1:**
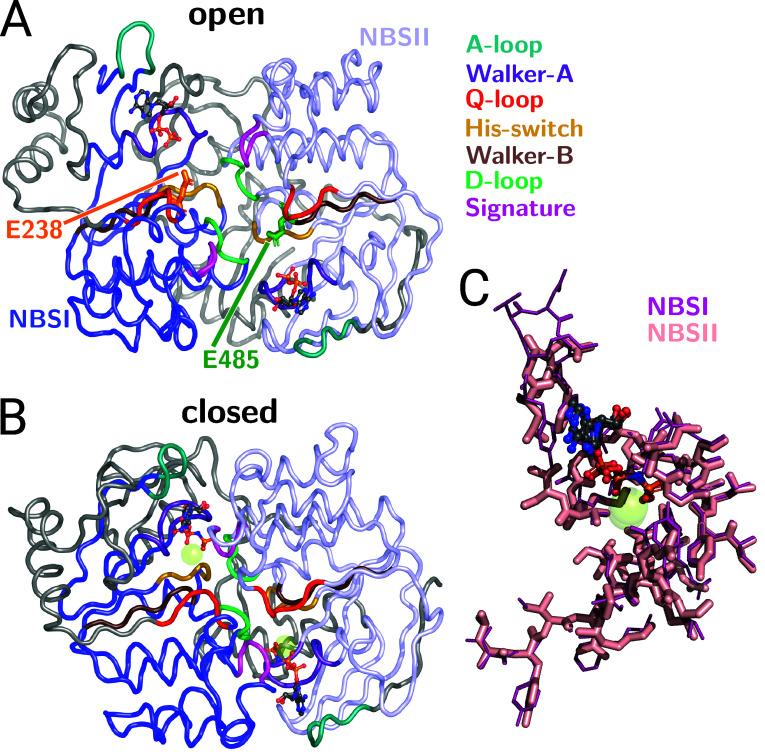
Structure of ABCE1 lacking the FeS domain. (A) Open conformation (pdb code 3BK7)^10^ and (B) closed conformation (pdb code 6TMF)^10^ in ribbon representation (NBDI: blue, NBDII: light blue, all other residues in gray) with bound ligands (ADP, ATP analog AMP-PNP, and Mg) in ball-and-stick representation. Regions referred to in the main text are indicated by color. (C) Selected regions of ABCE1 in its closed conformation with two bound AMP-PNPs molecules (pdb code 6TMF).^8^ Shown is a superposition of the two near symmetric NBSs NBSI and NBSII based on alignment of the seven highly conserved residues of each NBS.

As expected based on the high similarity of the NBDs, ABCE1 also hosts two highly similar ATP-hydrolysis-competent nucleotide-binding sites (NBSs), referred to as NBSI and NBSII. Both NBS are located at the interface between the two antiparallel-oriented NBDs and in fact comprise residues of both NBDs. A structural alignment and superposition of the two NBSs (Figure 1C) further illustrates the high similarity between both NBSs.

Multiple ABCE1 structures have been solved,^7–24^ which, taken together, suggest a ligand-dependent opening and closing motion of the dimer as the elementary chemo-mechanical process.^8^ The specific structure of the dimer further suggests that this opening and closing motion always involves both NBSs, i.e., the two NBSs are dynamically highly coupled such that one cannot be open while the other is closed.^25^ This feature will turn out to be crucial for our understanding of the molecular determinants of ABCE1 function.

In addition to the available structural information, ensemble and single-molecule experiments on ABCE1 have been carried out to characterize the thermodynamics and kinetics of ABCE1.^7,25,26^ In particular, to obtain information on the individual NBSs, two site-selective ATP-hydrolysis defective mutants were generated, and the ATP turnover rates of these mutants were measured and compared to the wild type.^7^ In each of the two mutants, the catalytically active Walker-B motif glutamate of one NBS was changed to glutamine, while that of the other binding site was left intact, resulting in drastically reduced ATP catalysis rates for the respective NBS. Mutant E238Q has the modification in NBSI and mutant E485Q in NBSII, allowing the ATP turnover rate of the opposite NBS to be measured separately in each case.

The high similarity between NBSI and NBSII would suggest that each NBS contributes equally to the wild-type ATP turnover rate, such that abolishing one of the NBSs while leaving the other intact should reduce the turnover by a factor of about two compared to that of the wild type. This reduction has indeed been observed for mutant E238Q; however, quite unexpectedly, mutant E485Q did not show similarly reduced ATP turnover. Instead, inactivating NBSII results in a 10-fold increased turnover rate! ^7,26^ Such pronounced asymmetry is, in fact, unprecedented among all members of the ABC superfamily. For example, MDR1a P-glycoprotein shows a quite symmetric decrease in ATP turnover rate for mutants with identical EQ point mutation in each NBS.^27^

It has been suggested that this striking asymmetry reflects different functions of the two NBSs. Whereas NBSI may control structural changes of the FeS domain resulting in ribosome splitting, NBSII has been suggested to act as a “timer” that triggers dissociation of the 30S ribosomal subunit after ATP hydrolysis, thereby terminating ABCE1 function in ribosome antiassociation.^26^

Clearly, such unexpected asymmetric kinetics calls for an explanation. A straightforward one is to assume a direct allosteric communication between both NBSs, such that ATP-binding in NBSII directly affects and facilitates ATP-binding or hydrolysis (or both) within NBSI.^8,25,26^ This hypothesis would explain the increased ATP turnover rate of mutant E485Q in terms of an increased ATP occupation of hydrolysis-defective NBSII and, thus, by an—on average—higher population of molecules with “activated” NBSI.^8^

In the following, we briefly summarize the support for this hypothesis by comparison with other members of the ABC superfamily with homologous NBDs, where some evidence indicates an allosteric pathway that facilitates direct communication between the two NBSs.

To investigate a possible allostery in the bacterial exporter Sav1866, a 150 ns molecular dynamics simulation was carried out starting from an AMP-PNP/AMP-PNP crystal structure (the notation gives the occupation of NBSI and NBSII, respectively).^28^ To mimic ATP unbinding, the NBS occupations were changed to ATP/apo, and the simulations resulted in a hydrolysis-competent state of NBSI.

Based on the simulations, the ATP unbinding from NBSII was suggested to be followed by a series of conformational rearrangements from the Walker-B motif of NBSII through the D-loop of NBDII to the Walker-A motif of NBSI.^28^ Although this simulation study suggests the possibility of direct allosteric communication, it has the reverse effect compared to what would be required for ABCE1, in that ATP binding in NBSII results in inactivation rather than activation of NBSI.

A similar communication pathway was proposed based on the comparison of apo/apo- and ATP/apo-bound crystal structures of bacterial exporter TM287/TM288, where binding of ATP to NBSI affects the stereochemistry of the Walker-A motif of NBSII.^29^ However, TM287/TM288 is a heterodimeric ABC exporter and, in contrast to ABCE1, has one NBS—termed degenerate or noncanonical—that lacks critical conserved residues for ATP hydrolysis, thus rendering the NBS ATP-hydrolysis incompetent. It is hypothesized that in transporters containing one canonical and one noncanonical NBS, the two sites have evolved to perform distinct functions, in contrast to transporters with two canonical sites, where both sites exhibit equivalent functionality.^30^ This functional divergence may result in differences in the communication between the NBSs in these distinct transporter types, potentially limiting the applicability of findings from such asymmetric transporters to ABCE1. Additionally, molecular dynamics simulations of TM287/TM288 showed no indication of allostery between the NBSs when started from the ATP/apo structure with ATP added to the second NBS.^31^ Detailed structure information under turnover conditions is also available for heterodimeric bacterial exporter TmrAB,^32^ albeit without indication of allostery, too.

Finally, in the DNA double-strand break repair protein Rad50, an alanine mutation of the D-loop aspartate resulted in reduced cooperativity of ATP hydrolysis,^33^ which indicates an involvement of the D-loop aspartate in the direct allosteric communication between the NBSs. However, neither wild type nor mutants of ABCE1 show any such cooperativity,^7^ rendering support along similar lines impossible.

In summary, and given these differences between the homologues, it seems to us that the evidence for—as well as against—direct allosteric communication between the two NBSs of ABCE1 is weak. In the absence of further support, here we ask: is it possible to explain the striking turnover rate asymmetry without recourse to any direct allosteric interactions, i.e., only through concerted opening and closing of the two NBSs? And if so, what is the underlying molecular mechanism? Besides the specific example of ABCE1, our main goal is to explore which mechanisms can possibly give rise to the observed strikingly asymmetric kinetics and, in particular, to such a dramatic turnover rate increase upon blocking a hydrolysis pathway, as observed for ABCE1.

To answer these questions, we describe the chemical reactions (ATP hydrolysis) and conformational transitions of ABCE1 as a Markov model. It has in fact been previously shown that chemical reaction networks are, under certain conditions, equivalent to Markov models.^34^ Recent examples are the use of Markov models to analyze translation,^35,36^ transcription,^37,38^ signaling pathways,^39^ molecular motors,^40–47^ and complex enzymes such as the fatty acid synthase.^48^ Accordingly, we describe the ABCE1 reaction cycle in terms of a set of discrete Markov states (Figure 2A), where each state represents a particular conformational (open or closed) and chemical (empty, ATP-bound, or ADP + P_*i*_-bound) state of ABCE1. Accordingly, transitions between these states represent conformational changes, ligand exchange, or ATP catalysis.^49^ In describing these transitions within a Markovian framework, we assume that they are memoryless, i.e., that all transition rates depend on only the current state. Note that we do not use Markov models as a coarse-graining of otherwise high-dimensional atomistic descriptions such as molecular dynamics simulations,^50–52^ but rather we design the Markov models using an empirical, top-down approach.

**Figure 2:**
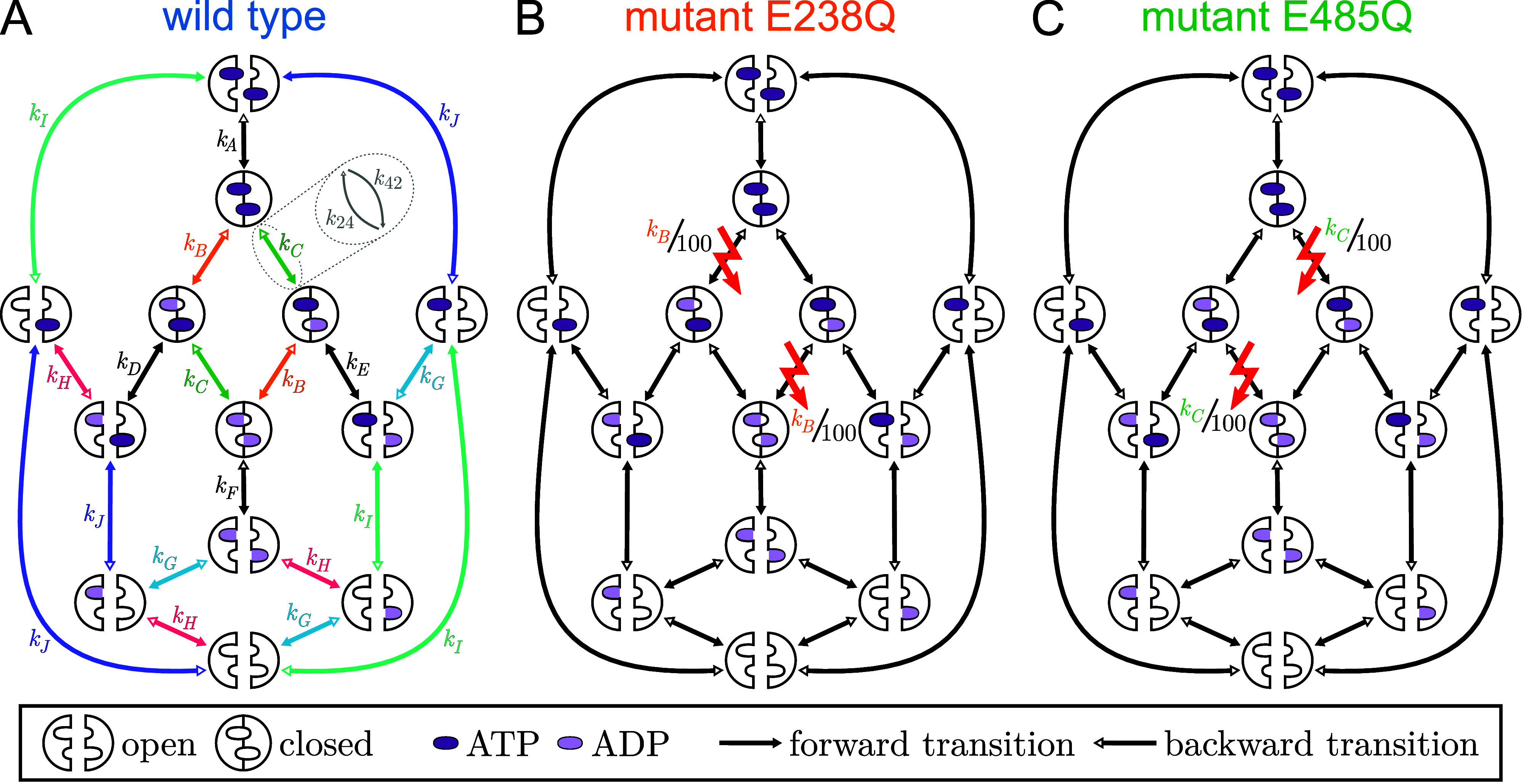
Graph representations of ABCE1 Markov model classes (A) wild type, (B) mutant E238Q, and (C) mutant E485Q. Each ABCE1 symbol represents one Markov state that is connected to other states by conformational or chemical transitions (arrows) with indicated transition rate coefficients *k*_*i*_. In (A), the absence of any direct allosteric interaction between the NBSs is implemented by assuming equal transition rate coefficients for the transitions indicated by identical colors and labels (*k*_A_, *k*_B_, *k*_C_..., see also Table S1). All transitions are reversible; for clarity, only the forward rates (solid arrow heads) are annotated. For the two mutants, the reduced ATP hydrolysis rate coefficients of the respective defective NBS are indicated by the red lightning symbols; all other transition rate coefficients are assumed identical to the wild type. Filled dark purple ovals denote bound ATP; partially filled light purple ovals denote bound ADP, indicating the one missing phosphate; for clarity, inorganic phosphate is not shown.

Such coarse grained yet thermodynamically consistent description^48,53^ enables us to define the Markov model of ABCE1 such that any direct allostery between the NBSs is excluded by construction. If such a model is nevertheless capable of reproducing the asymmetric turnover rates observed for the wild type and both mutants, one would conclude that indeed, allostery is not required to explain this asymmetric ABCE1 kinetics. Of course, such a result would not rule out direct allosteric interactions between the two NBSs either.

To this end, we proceed as follows: First, we only define the “topology” of the Markov model, i.e., its states and its connections. We refer to this model level, which does not include specification of transition rate coefficients between the Markov states, as a Markov model class. Each Markov model class, therefore, comprises many specific Markov models, which are defined, in addition, by their transition rate coefficients. Hence, we ask if Markov models of this Markov model class can be identified that agree, within experimental uncertainty, with the measured ATP hydrolysis kinetics.

To assess whether such models exist, we employ Bayesian inference to determine the probability of each Markov model given the available measurements.^54^ Should many such Markov models with non-negligible probabilities be found, we will group them into sets representing similar molecular mechanisms. In contrast to previous maximum-likelihood based approaches,^39,40,48,55^ this Bayesian approach will also enable us to rank different possible mechanisms according to their probability.

## 2 Results

To test the hypothesis that no direct allosteric communication between the two NBSs of ABCE1 is required to explain the asymmetric ATP hydrolysis kinetics of the two mutants, we describe the combined conformational dynamics, ATP binding and hydrolysis, and product unbinding as a Markov model, which by construction does not contain such a direct interaction. Therefore, if the observed peculiar and counterintuitive ATP hydrolysis kinetics can be reproduced by the Markov model, we can conclude that these kinetics can be explained without direct allostery.

Note that we aim to explain the asymmetric ATP hydrolysis kinetics as measured by Barthelme et al.^7^ on free ABCE1 in the absence of ribosomal subunits and further translational factors. Thus, our models of ABCE1 will also describe free ABCE1 and do not include any ribosome-bound states of ABCE1.

### 2.1 Markov Model Class of ABCE1

First, we define the states and transitions without specifying particular transition rate coefficients between states. We will refer to this definition as a “Markov model class”, and instances of that class with specified transition rate coefficients will be referred to simply as “Markov model”. Our goal is to design a Markov model class of ABCE1 that is, on one hand, minimal in terms of number of states and transitions (to keep the number of parameters low) and, on the other hand, complex enough to capture the asymmetry of ABCE1. Furthermore, we aim at a Markov model that is thermodynamically consistent, i.e., that all relevant states are considered, that the concentrations of ATP, ADP, and P_*i*_ are properly taken into account, and that detailed balance is obeyed. Detailed balance requires that all of the transitions are bidirectional.

Figure 2A shows the Markov model class of the wild type. Here, we used the following assumptions to determine its number of states, their association with conformational and occupational states of ABCE1, and the transitions between them.

First, we assumed that two conformational states—an open and a closed state—of the NBSs suffice to describe ATP hydrolysis of free ABCE1. These two states are well-known from X-ray structures of free ABCE1 occupied by ADP showing ABCE1 with NBSs in an open conformation^7,9,10^ and from cryo-EM structures of ABCE1 bound to the small ribosomal subunit showing ABCE1 with NBSs in a closed conformation.^8,14,56^

In addition to the open and the closed state, cryo-EM structures of presplitting 70S-ABCE1 and 80S-ABCE1 complexes show the NBSs of ABCE1 in an intermediate or half-open conformation.^11,12,16–20^ Additional support for such an intermediate state of NBSs comes from FRET efficiency distributions of dyes attached to the two NBD-parts of each NBS, which suggest at least three distinct states.^25^ On a functional level, the intermediate state of ABCE1 is considered necessary for regulation of ribosome separation.^8^ In the absence of evidence of a role of this intermediate state in the ATP hydrolysis of free ABCE1, we tentatively assumed that an intermediate third conformational state is not required to explain the kinetic asymmetry of the mutants.

Second, we assumed that both NBSs adopt the same conformation at all times—either both are closed or both are open. This assumption is supported by the fact that all free, 80S-bound, and 30S-bound ABCE1 structures known to us show both NBSs in identical conformations.^7–12,14–21,24^ We note that cryo-EM structures of 43S and 48S late initiation complexes show NBSI in the intermediate conformation and NBSII in the closed conformation bound with GMP-PNP/GMP-PNP^21,24^ or ADP/ATP,^22,23^ respectively. Also, differences in FRET efficiencies corresponding to the two NBSs indicate a certain degree of conformational independence of the NBSs,^25^ but no direct measurement of the correlation between the conformational states of the two NBSs exists to date. Whereas such evidence may suggest a certain degree of conformational independence between the NBSs, we decided to first test if the asymmetric ATP hydrolysis kinetics can be reproduced without assuming such independence.

Third, we assumed that ABCE1 closes only if both NBSs are occupied by a nucleotide. Indeed, all known closed structures^8,14,56^ and intermediate/closed structures^13,21–24^ of sufficient resolution do show nucleotides in both NBSs. Additional experimental evidence comes from mutants of ABCE1 that are unable to bind nucleotides in one or both NBSs.^26^ These mutants are also unable to split ribosomes, which requires closing of both NBSs,^8,14,56^ and, consequently, binding of two nucleotides is necessary for ABCE1 to adopt a closed state and split ribosomes.^26^ Further, in the absence of nucleotides, FRET-experiments showed that NBSII remains in an open conformation.^25^ This assumption led us to omit closed conformations with empty NBSs from our Markov model class and, furthermore, reduced the number of transitions between Markov states representing open and closed conformations of ABCE1.

The next two assumptions define the transitions between Markov states of equal conformation.

The fourth assumption is that ligand exchange occurs only in the open conformation, which is sterically plausible because X-ray structures^7,9,10,15^ of the open conformation show that the distance between both NBDs, termed nucleotide-binding cleft, is large (10 Å–14 Å).^10^ In contrast, in all structures of ABCE1 in the closed state, two AMP-PNP are trapped between the two NBDs keeping them in close proximity without notable nucleotide binding cleft.^8,14^

The fifth assumption is that ATP hydrolysis occurs only in the closed conformation, which, too, is sterically plausible from structures because, in the closed conformation, all residues required for ATP hydrolysis are in contact with bound AMP-PNP.^8,14^ In the closed state, the signature motif and D-loop of the opposite NBD complement the binding site and coordinate the γ-phosphate. In the open conformation, the large nucleotide-binding cleft positions these two hydrolysis motifs too far from a bound ATP to establish interactions required for ATP hydrolysis.^7,9,10,29,57^ As a result, ATP hydrolysis is inhibited in the open conformation.

The sixth assumption is that there is no allosteric communication between the NBSs. This is the central assumption to test our hypothesis that this sort of allostery is not necessary for the asymmetric ATP hydrolysis kinetics of ABCE1 mutants. On a technical level, this means that the transition rate of one type of transition, e.g., ATP binding to NBSII, is independent from the occupation of the opposite NBS, and thus, these rates occur multiple times in the model as indicated by identical labels *k*_A...J_ in Figure 2A.

The last assumption, which is more technical in nature, aims at reducing the complexity of the Markov model. To this aim, we assume that ADP- and P_*i*_ exchange can be described by only one effective transition, which implies that the respective on- and off-rates describe the long-time scale kinetics of the two subsequent binding and unbinding events. Thus, our model does not distinguish between ADP-, P_*i*_-, and ADP + P_*i*_-bound states and considers for each conformational state and each NBS of ABCE1 an empty, an ADP + P_*i*_-bound, and an ATP-bound binding state. Accordingly, we will henceforth refer to ADP and P_*i*_ exchange collectively as the ADP exchange. This assumption is consistent with the most recent model of ABCE1-driven ribosome separation, which also combines ADP and P_*i*_ release into one transition.^8^ Furthermore, this assumption will turn out to be computationally beneficial, as it reduces the number of distinct combinations of occupations from 5^2^ (apo, ATP, ADP + P_*i*_, ADP, and P_*i*_) to 3^2^ (apo, ATP, and ADP), thereby reducing the number of Markov states—and, hence, the dimension of the search space—by over 2-fold.

Recent structural studies of bacterial exporters TmrAB^32^ and TAP1/2 ^58^ have shown that in these transporters, P_*i*_ dissociates from the protein prior to the opening of the NBSs and the subsequent release of ADP. If this mechanism is also applicable to ABCE1, our results would remain valid, albeit with a revised interpretation of the transitions. In this case, the effective transition for ADP + P_*i*_ binding is unchanged, while the ADP + P_*i*_ unbinding transition would be redefined as only ADP unbinding. The current opening transition would then also encompass P_*i*_ release.

Taken together, the above assumptions result in the Markov model class of ABCE1 shown in Figure 2A with 13 states and 10 forward and 10 backward transition rate coefficients. Table S1 lists all transition rate coefficients along with the corresponding conformational changes, chemical reactions, and ligand exchanges. These transition rate coefficients are unknown and were determined via Bayesian inference as described below.

The Markov model classes of mutants E238Q and E485Q (Figure 2B,C) are derived from the wild-type class by reducing the transition rate coefficients of ATP hydrolysis (*k*_B_ and *k*_C_) and synthesis (*k*_–B_ and *k*_–C_) of the mutated NBS by a factor of 100. This reduction describes the fact that the mutation only renders the NBS ATP-hydrolysis defective rather than completely blocking it.^7,26,27^ Further, this reduction implements as an eighth assumption that the point mutations affect only the ATP catalysis of the respective NBS, leaving catalytic rates and binding affinities of the other NBS unaffected.

Finally, we incorporated detailed balance into the Markov models, which reduces the number of parameters by three.^59^ Rather than implementing proper restrictions on the transition rate coefficients, however, we derived the latter from assigning a free energy to each Markov state, which we consider to be more intuitive and straightforward. As a result, our Markov models are parameterized by 17 parameters, four free binding energy differences (ATP and ADP binding for each NBS), one free energy difference associated with the conformational change, two free energy differences between ATP and ADP bound in the closed state (one for each NBS), and one free energy barrier for each transition A,B,···,J (see Figure 2A). To convert between free energies and transition rate coefficients, we used transition state theory,^60^ which states that

1 $$k_{ts}=\omega_{0}e^{-\Delta G_{ts}^{‡}}$$

where *k*_*ts*_ is the transition rate coefficient from state *s* to state *t*, Δ*G*_*ts*_^‡^ is the free energy barrier between state *s* and state *t*, and ω_0_ is the attempt frequency. Here, the particular choice of ω_0_ is irrelevant, as any change of ω_0_ can be absorbed into the barrier heights; to allow for intuitive interpretation of the barrier height, we have chosen $\omega_{0}=\frac{\kappa k_{B}T}{h}$ with κ the transmission coefficient (hereafter set to one), *k*_B_ the Boltzmann constant, *T* the temperature, and *h* the Planck constant.

These Markov models will subsequently serve to calculate and compare the measured kinetic observables, i.e., limiting ATP turnover rate and Michaelis–Menten constant.

### 2.2 Markov Models Reproduce Asymmetric ATP Hydrolysis Kinetics

To determine whether Markov models exist that agree with the measured ATP hydrolysis kinetics,^7^ we used Bayesian inference. Specifically, for each Markov model M, its posterior probability was calculated via

2 $$P\left(M|\left\{\mu_{i},\sigma_{i}\right\}\right)\propto P\left(\left\{\mu_{i},\sigma_{i}\right\}|M\right)P\left(M\right)$$

for the six measured values μ_*i*_ (3 ATP turnover rates and 3 Michaelis–Menten constants for wild type, mutant E238Q, and mutant E485Q) and their respective experimental uncertainties σ_*i*_. Here, the σ_*i*_ are not nuisance parameters, as might be assumed; instead, these are experimental uncertainty estimates^7^ and thus are fixed parameters of the posterior, eq 2. Assuming a Gaussian error model for the experiments, the likelihood^54^ reads

3 $$P\left(\left\{\mu_{i},\sigma_{i}\right\}|M\right)=\prod_{i=1}^{6}N\left(x_{i}|\mu_{i},\sigma_{i}\right)$$

where N is a normalized Gaussian and *x*_*i*_ is the kinetic observable as calculated from the specific Markov model under consideration. As prior *P*(M), a product of uniform priors of the free energies was chosen with physics-motivated upper and lower limits depending on the type of the particular transition (see Methods). This choice of the prior implies a log-uniform prior for the respective transition rate coefficients and transition times. For posterior sampling a Markov-chain Monte Carlo algorithm^61^ was used with multiple chains to assess the convergence of Bayes-sampling.

To check whether the obtained distribution of Markov Models results in the expected distribution of experimental observables centered at the respective measured values, all six kinetic observables were calculated for each Markov Model (see Methods) and are shown as histograms in Figure 3. As can be seen, both the limiting ATP turnover rate *k*_cat_ (Figure 3, top row) and Michaelis–Menten constant *K*_M_ (Figure 3, bottom row) agree well with the measured values and are distributed following the experimental uncertainties σ_*i*_, indicating correct and sufficiently converged Bayes-sampling of the Markov models.

**Figure 3:**
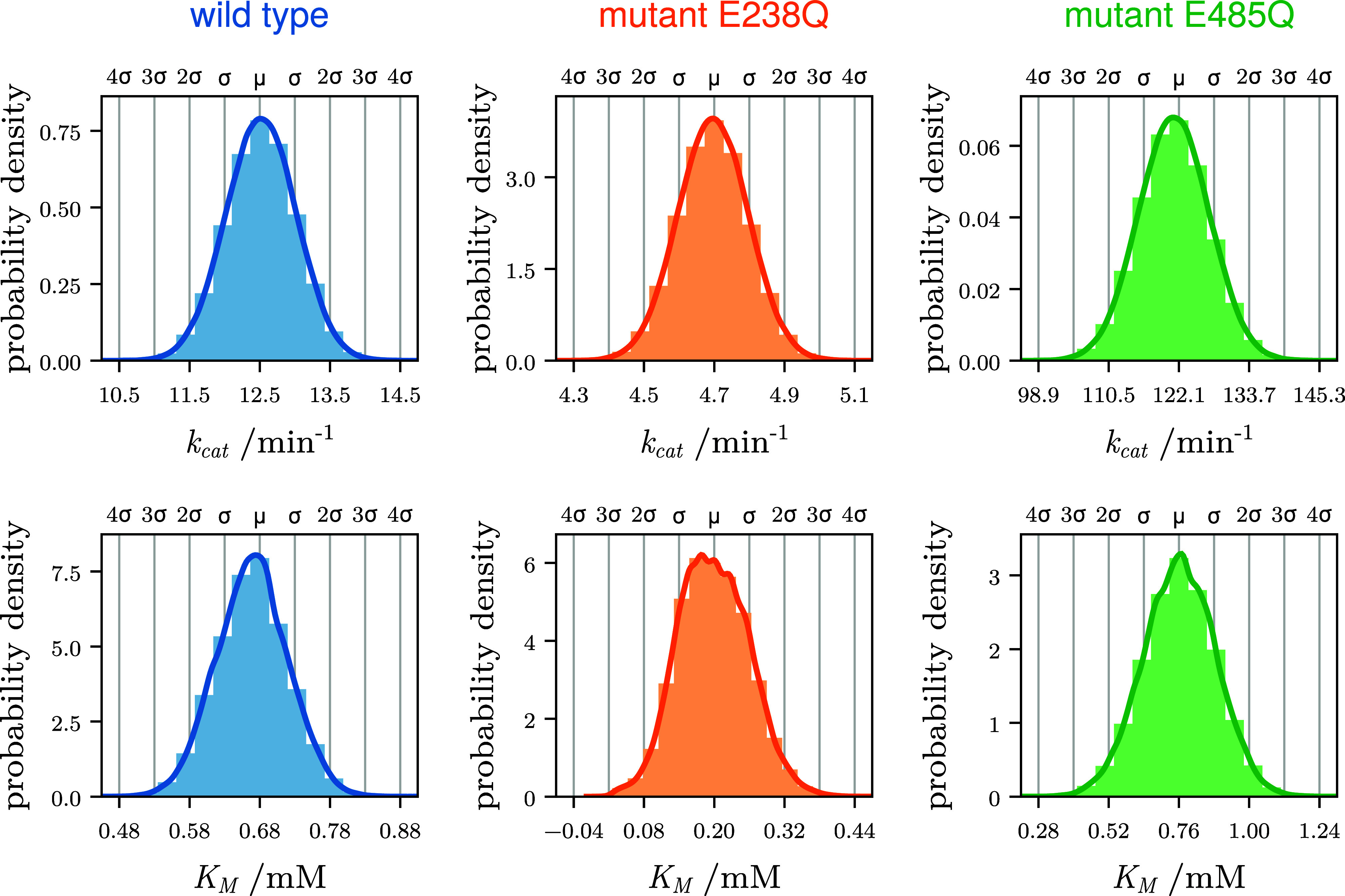
Posterior probability distributions of Michaelis Menten constants. Shown are histograms and fitted kernel densities (solid lines) of *k*_cat_ (top row) and *K*_M_ (bottom row) for wild-type (left), E238Q (mid), and E485Q (right), as obtained from Bayes-sampling of the respective Markov models. The annotations on the top of each plot show the measured values^7^ μ and experimental uncertainties (in units of standard deviation σ).

A sufficiently converged Bayes-sampling is further supported by major overlap between multiple Markov-chain Monte Carlo runs for all free energy parameters with minor deviations of individual runs, for example, for barrier Δ*G*_E_^‡^ of the conformational change with ATP/ADP occupations for values ≳38 kT (see Figure S1).

Next, we asked whether there are any Markov models that agree with all six measured kinetic observables and, if so, how many. We found that 9.3% of all sampled Markov models agree with all six measured observables within experimental uncertainty σ_*i*_ and, hence, reproduce the observed striking asymmetry.

We conclude that at least within our Markovian framework, no direct communication between the two NBSs is required.

### 2.3 Lopsided Wild-Type Populations Enable Asymmetric ATP Hydrolysis Kinetics

Next, we investigated the mechanism by which the asymmetric ATP hydrolysis kinetics arises in our Markov models. To this end, we identified in each Markov model those reaction cycles that contribute most to the ATP hydrolysis. Here, reaction cycles are defined as closed sequences of Markov states which involve one hydrolysis step, and the contribution ϑ to ATP hydrolysis was quantified by the probability net flux Θ, i.e., the difference of population multiplied by the transition rate coefficient between two states, through the respective cycle relative to the total ATP hydrolysis rate of that Markov model. For instance, the sequence of states “1–2–3–5–12” corresponds to the hydrolysis of ATP in NBSI with ATP bound in NSBII.

We used these contributions to classify all sampled Markov models that agreed with all measurements within experimental uncertainty into “reaction types”. To this end, we considered only “dominant” reaction cycles with ϑ above a threshold of 20% at saturating concentrations of ATP and grouped them accordingly. Importantly, the Bayesian sampling according to the posterior model (eq 2) enabled us to calculate the probability of each reaction type as the normalized count of Markov models of that type.

Further support for this grouping comes from an analysis of the distributions of net fluxes separated by ATP hydrolysis transitions. As shown in Figure S2A, most of these distributions are multimodal, and our grouping into reaction types decomposes them into monomodal distributions (Figure S2B–D).

Strikingly, a total of almost 500 different reaction types were determined, highlighting the complexity of a complete reaction kinetics analysis of even such a comparably simple biomolecule. Figure 4 shows one randomly selected example for each of the three most probable reaction types (“A1”, “A2”, and “B”), which, taken together, account for 40.4% of all sampled Markov models and, hence, deserve closer analysis. In Figure 4, the net flux Θ is indicated via the width and color of each arrow (transition), and the size and color of each state (vertex) indicates the steady-state probability, i.e., the fraction of enzymes expected to be in each state, henceforth referred to as the population. Dominant reaction cycles are drawn solid, and all others are dashed.

**Figure 4:**
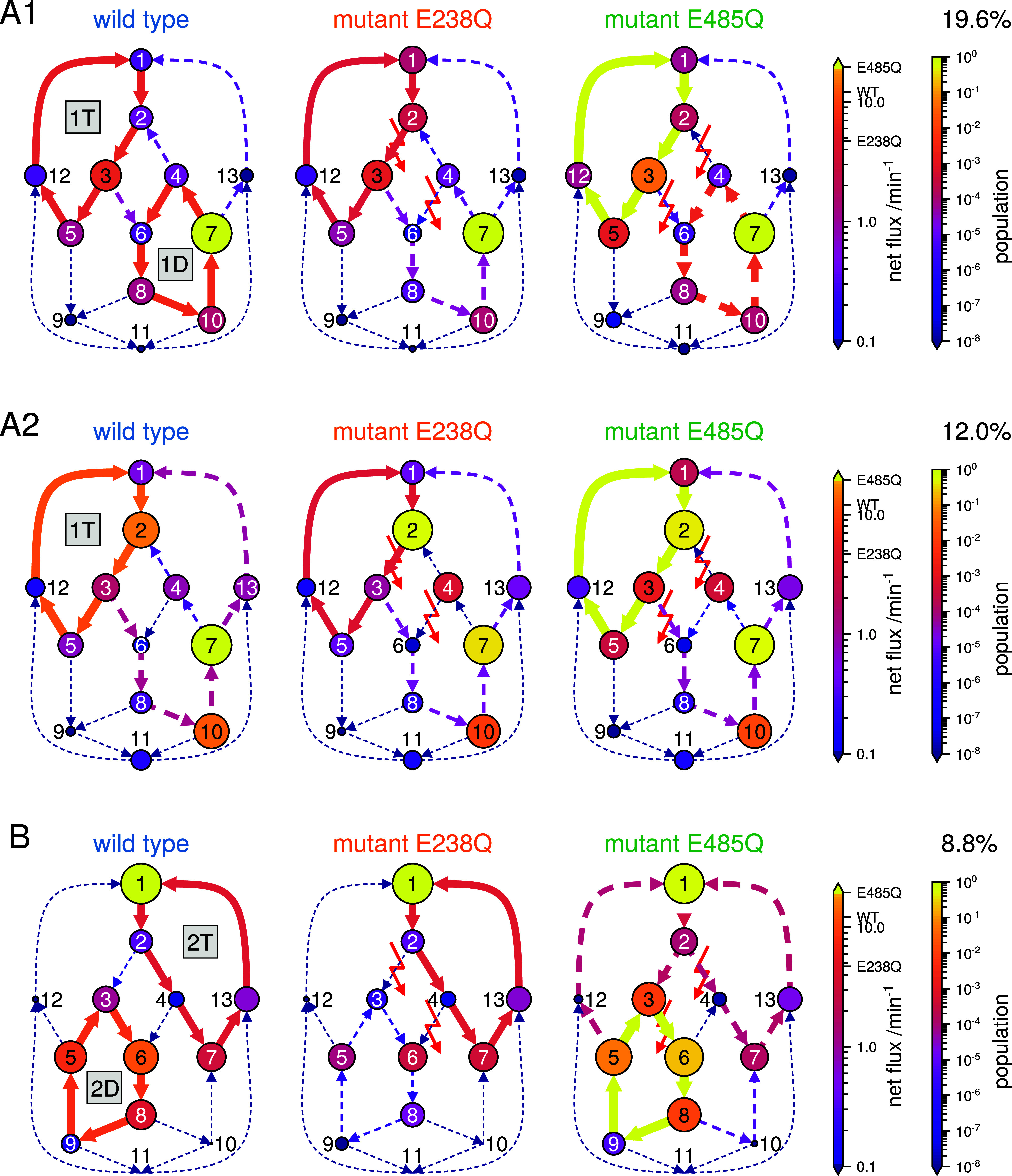
Three most probable reaction types. The posterior probability for each reaction type A1, A2, and B is indicated as a percentage. Markov states (circles) are defined as in Figure 2, and their populations are indicated by size and color; similarly, the net fluxes through the transitions (arrows) are indicated by line widths and colors. Dominant reaction cycles (defined in the text) are labeled (“1T”, “1D”,···) and are indicated by solid lines, otherwise dashed. For the two mutants, the reduced ATP hydrolysis rate coefficients of the respective defective NBS are indicated by the red lightning symbols.

We use the most probable reaction type A1 (Figure 4, top row) to explain a plausible mechanism underlying the asymmetric ATP hydrolysis kinetics (refer also to Figure 2). In this reaction type, mainly two cycles contribute to the overall ATP turnover rate in the wild type. In the upper left cycle (denoted by “1T”), hydrolysis occurs in NBSI, while NBSII is ATP-loaded. In the lower right cycle (“1D”), hydrolysis also occurs in NBSI, with NBSII being ADP-loaded. Thus, unexpectedly, in the dominant reaction type A1, by far most of the ATP hydrolysis is performed by NBSI. Although the net fluxes of cycles 1T and 1D are similar, their kinetics is very different. Compared to cycle 1T, the population 1D states is much (100–1000×) higher, which is compensated by lower rates. In fact, almost all of the total population of the system is concentrated in state 7, i.e., ABCE1 is open most of the time, with NBSI loaded with ATP and NBSII with ADP. As will become important below, this population asymmetry between the two cycles is largely controlled by the ATP catalysis rates of NBSII, which creates a net flux from state 3 to 6 and thus acts as a “drainage” of cycle 1T into cycle 1D. As the E485Q mutation hinders ATP catalysis in NSBII, it drastically reduces this drainage. Overall, the observed ATP hydrolysis rate does not simply result from one reaction cycle but arises from a combination of several cycles and a fine-tuned population balance between these.

This balance is markedly shifted by the E238Q mutation, which eliminates ATP hydrolysis in NBSI almost entirely (Figure 4, top middle). Despite this 100-fold reduction of NBSI, cycle 1T is still active, and NBSI is still the main contributor to the overall ATP turnover rate. This counterintuitive response is explained by an increased population of states upstream of ATP hydrolysis (states 1 and 2), where both NBSs are ATP-loaded. Essentially, this population “piles up” due to the subsequent larger barriers. The strength of this particular effect—as well as precisely which upstream states are being affected—depends on the particular choice of the individual transition rate coefficients. In contrast, cycle 1D is largely suppressed. Here, the population of upstream states drains from state 4 into state 2, further contributing to the population increase of upstream states of cycle 1T, thus contributing to the only slight decrease of cycle 1T and the 2-fold ATP turnover rate.

The mutation E485Q, in contrast, does not directly affect transitions within the 1T and 1D cycles but the transitions responsible for the drainage between cycles. As a result, the drainage of cycle 1T via hydrolysis in NBSII is largely suppressed (Figure 4, upper right), resulting in a ca. 10- to 20-fold higher population of this cycle. It is this drastic population shift that causes the nearly 10–20-fold increase of the ATP turnover within cycle 1T, despite its transition rate coefficients being unchanged relative to the wild type.

The second most probable reaction type, A2 (Figure 4, middle row), is very similar to the reaction type A1. The main difference is that cycle 1T is the sole dominant contributor to the overall ATP turnover rate in the wild type. Still, as with reaction type A1, cycle 1T is not highly populated in the wild type, enabling a large population shift in mutant E485Q, when the drainage from cycle 1T is largely suppressed.

For the third most probable reaction type (Figure 4, bottom row), the mechanism is similar to reaction type A1, but with cycle “2D” taking over the role of 1T and cycle “2T” that of 1D. As a result, the effects of the mutations are also switched. In particular, in reaction type B, mutant E238Q now impacts the drainage between the cycles, whereas mutant E485Q directly impacts cycles 2D and 2T. However, the switched roles still lead to the same effect of the mutations on the overall ATP turnover. For mutant E238Q, and in contrast to mutant E485Q in reaction types A1 and A2, no large population shift is observed because most population is already located in source cycle 2T of the drainage. Instead, this drainage (from cycle 2T to cycle 2D) is interrupted, resulting in suppression of cycle 2D, thereby halving the overall ATP turnover rate. For mutant E485Q, an increase in the upstream states (3 and 5) of ATP hydrolysis in NBSII is observed, similar to that of mutant E238Q in reaction types A1 and A2. However, the effect is even stronger due to the particular choice of the individual transition rate coefficients.

Next, we asked what causes the asymmetric population distribution between the cycles. To answer this question, Figure 5 shows, for each Markov state separately, the population distribution of all sampled Markov models, ignoring their particular reaction type. Clearly, for the wild type, for over 90.6% of all sampled Markov models, one of the states 1, 6, or 7 is by far the most populated one (≥90% population), leaving only very low populations for all other states.

**Figure 5:**
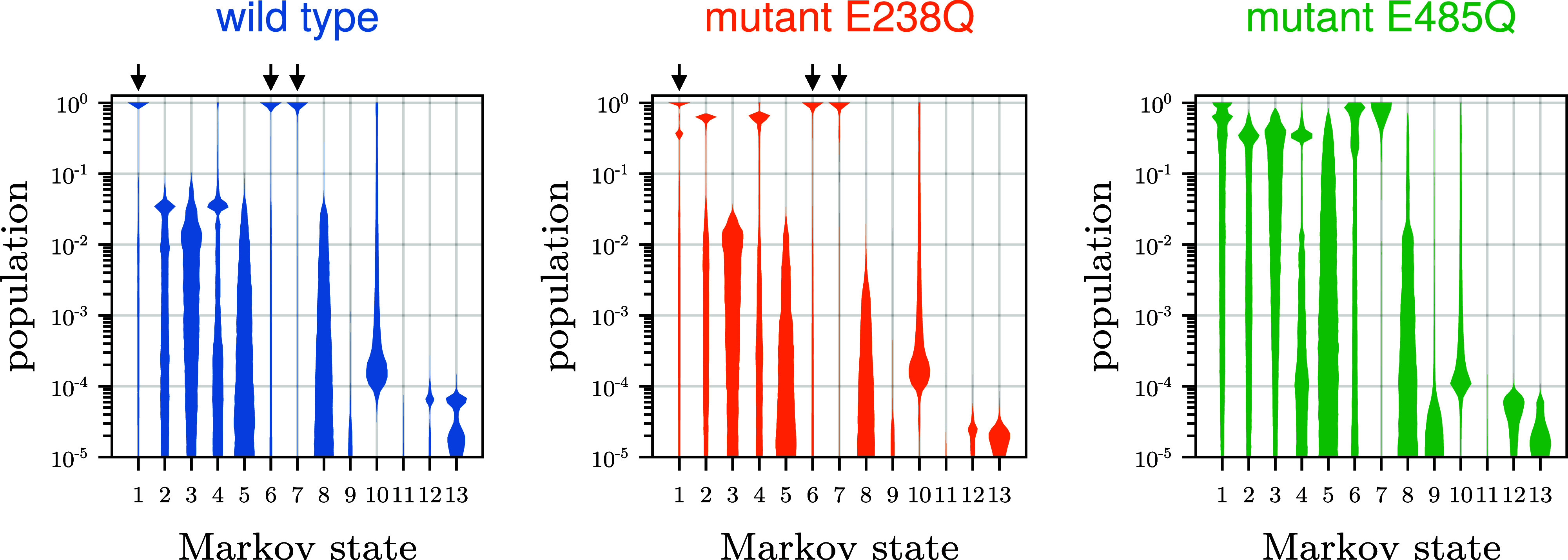
Posterior probability distributions of populations for each Markov state. Shown are distributions of the steady-state populations at conditions of limiting ATP-turnover rate ([ATP] = 100 mol L^–1^) for wild type (left), mutant E238Q (mid), and mutant E485Q (right). Distributions are based on Markov Model samples of posterior 2. Arrows highlight Markov states in which population is concentrated.

This finding is remarkable because it is not obvious why concentrating nearly all populations in one Markov state should be required to achieve the observed ATP turnover asymmetry between the two mutants.

We speculate that the prevalence of Markov models with population dominance may be due to an “entropic factor”, i.e., that for a single state dominated Markov model, a larger region of parameter space yields agreement with the experimental data than for Markov models with several markedly populated states. Accordingly, since that latter may require more stringent fine-tuning of their parameters, single state dominated Markov models would be “easier” to find during sampling and may also be more robust. Such robustness of single state dominated Markov models has interesting evolutionary implications.

What property singles out states 1, 6, and 7 from all other states? Are these Markov states perhaps rate limiting for the overall turnover rate such that the flux through the network is largely dictated by the population of these states? To test this idea, Figure 6A shows the conditional probability for each Markov state to be the most populated one given the rate-limiting Markov state. Here, following the usual convention,^62^ the rate-limiting state was defined as the state for which a change of free energy has the largest effect on the overall ATP turnover rate (i.e., the highest degree of “thermodynamic” rate control). Indeed, in all cases, the most populated state is also the rate-limiting state of the Markov model.

**Figure 6:**
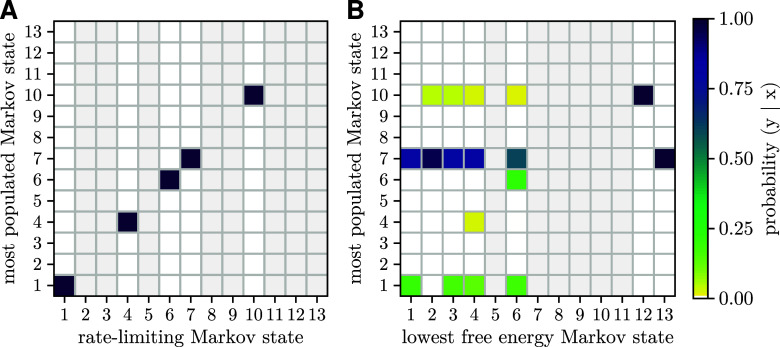
Comparison of the characteristics of wild-type Markov states. Shown are the conditional probabilities (color) of each Markov state to be the most populated, given either (A) the rate-limiting Markov state or (B) the Markov state with the lowest free energy. The column is left empty (gray) for states that were not a rate-limiting or lowest free energy Markov state in any of the Markov models.

To assess whether this agreement can be attributed to the thermodynamic properties of these states, i.e., whether the most populated state corresponds to the lowest free energy state, Figure 6B shows, in a manner similar to that of Figure 6A, the conditional probability for each Markov state to be the most populated one, now given the lowest free energy state. As can be seen, and in contrast to the rate-limiting state, almost no correlation is seen between the most populated Markov state and the lowest free energy state. This finding strongly supports the notion that the most populated states are highly enriched due to “kinetic traps” and thereby enable relatively high turnover rates despite slow transition rates.

To obtain further insight into why a particular state is the rate-limiting or most populated state, we also computed rate-limiting transition states^63^ and transition rate coefficients (commonly termed sensitivity^64,65^ ). However, as shown in Figure S3, no marked correlations were found between the most populated state and the rate-limiting transition state. Furthermore, while a transition rate coefficient of an outgoing transition of the most populated state is the rate-limiting transition rate coefficient in 94.8% of our Markov models, the remaining 5.2% show that this is neither a necessary nor sufficient condition for determining the most populated state. Both analyses underscore that these “kinetic traps” are due to a more complicated balance between several transition rate coefficients.

On the more technical side, two cautionary notes are in order. First, because the exact definition of “dominant reaction cycles” depends on the particular choice of a threshold (chosen above as 20%), so does the above classification into reaction types. Figure S4 quantifies this dependence and the resulting pattern of dominant cycles (insets). As can be seen, the three reaction types singled out (left insets) and discussed above dominate for a rather wide range of threshold values between 0.05 and 0.25, hence providing justification for their choice. For larger threshold values, Markov models with multiple reaction cycles of similar net flux are characterized by the absence of any dominant reaction cycles. This insensitivity means that models with different reaction cycles are classified into the same reaction type (two top right insets) such that they are not properly distinguished.

Second, it is unknown how much the ATP catalysis rate is reduced by the mutant. Our choice of a factor of 100 is motivated by ATP turnover measurements,^7,26,27^ which most likely provide an upper bound. To assess the robustness of our findings against this uncertainty, we changed this reduction factor by 1 order of magnitude in both directions and repeated the above Bayesian posterior sampling and reaction type identification protocol. Similar results were obtained; in particular, for a 1000-fold reduction, the same most probable reaction types (B, A1) are seen. For a 10-fold reduction, the kinetic asymmetry was qualitatively reproduced, although less pronounced in all sampled Markov models, and reaction type A1 was the most probable reaction type (99.9%). In summary, these results demonstrate that the reaction type that we identified as the most probable one is robust against the experimental uncertainty of how much the E238Q and E485Q mutations actually reduce the ATP catalysis rates.

In addition to direct allosteric interaction and although the two NBSs are rather similar to each other, their small differences may also contribute to the striking kinetic asymmetry. Here, the most notable difference of NBSII compared with NBSI is the presence of a leucine instead of an aromatic residue in the Walker A motif. Otherwise, the two structures are rather similar, including the positioning of bound ATP. Hence, one would not assume this asymmetry of the two NBSs to be a major factor. Indeed, replacing the leucine with a tyrosine did not alter ATP hydrolysis activity.^26^ In addition, three charge differences exist in the highly conserved motifs, but they are far from the bound nucleotides and are therefore not expected to impact binding and hydrolysis markedly. To identify possible remaining differences between the two NBSs that might explain the kinetic differences, Figures S6–S8 show the transition rate coefficients and free energies of the two NBSs for the three most probable reaction pathways. In reaction pathways A1 and B, the NBS mainly responsible for ATP hydrolysis in the E485Q mutant has a faster ATP hydrolysis and ADP unbinding transition rate coefficient in 100% of the Markov models, and in reaction pathway B, this is the case for around 80% of all Markov models. These results suggest that differences in these two transition rates between the two NBSs are the most promising candidates for modulating the observed kinetic hydrolysis asymmetry. For the homologous transporter BtuCD-F, it has been shown that ATP hydrolysis turnover in ABC proteins strongly depends on water coordination,^66^ an aspect that has not yet been investigated in ABCE1. Specifically, neither the crystal structures that show ABC in an open conformation with ADP bound^7,9,10^ nor cryo-EM structures of the closed conformation resolved the relevant water molecules,^8,14,56^ so that their role in NBS asymmetry remains unclear.

As a more technical note, we wanted to know to what extent the dependence of the ATP turnover rate on the ATP concentration in our Markov models (i.e., their turnover curves) follow Michaelis–Menten kinetics. To this end, Figure 7 compares turnover curves calculated from a randomly selected subset of our Markov models with measured ones.^7^ More than half (ca. 53%) of all Markov models indeed follow Michaelis–Menten kinetics quite closely. Because our Markov models are much more complex than the simple two-state Markov models from which Michaelis–Menten kinetics is derived and since we did not enforce Michaelis–Menten-like behavior in any way (see Methods for the heuristic approach of determining *K*_M_ and *k*_cat_), this result is quite unexpected. As a result, we also abstained from enforcing Michaelis–Menten-like turnover curves a posteriori.

**Figure 7:**
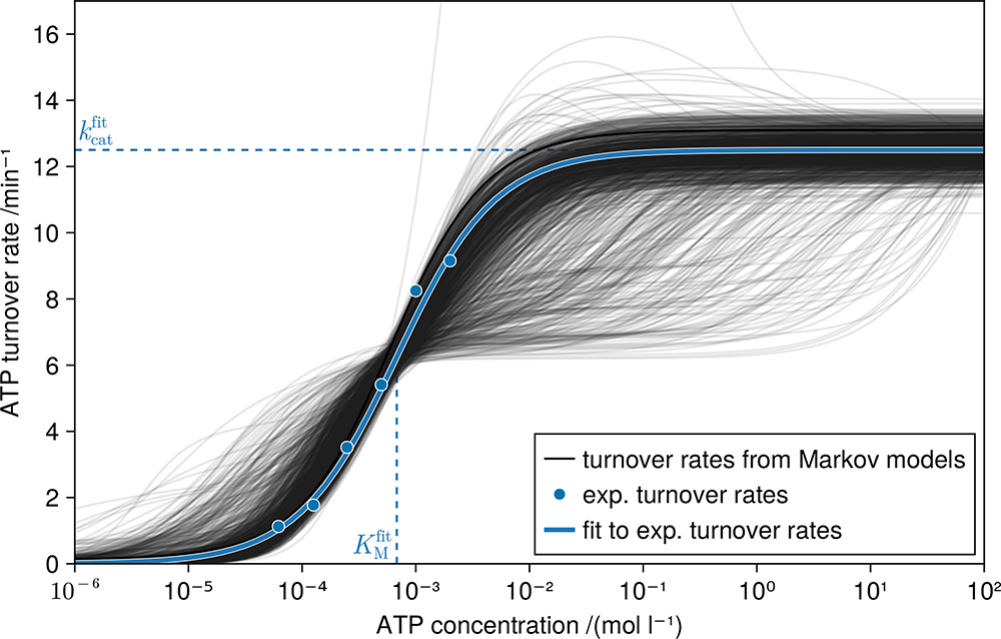
ATP turnover curves of wild-type Markov models. For 1049 randomly selected Markov models, the dependence of the wild-type ATP turnover rate on the ATP concentration are shown as gray lines. For comparison, the measured turnover rates and the fitted Michaelis–Menten kinetics curve^7^ are shown in blue.

### 2.4 Cross-Validation with Ligand Occupancies

To further test our Markov models and, in particular, their underlying physical models and assumptions, we checked how well the Markov models singled out so far agree with new experimental data that have not been used for calculations of the posterior and, hence, for the Bayes-sampling. To this end, we used measured ATP and ADP occupancies, for which two different experimental data sets are available—one taken under steady-state conditions^7^ and one measured under nonequilibrium/single-turnover conditions.^26^

Figure 8 shows the steady-state ATP occupancy measured by Barthelme et al.^7^ as red lines, together with the posterior ATP occupancy probability density derived from the Bayesian Markov sample, assuming a steady state. (The joint posterior ATP and ADP occupancy probability densities are shown in Figure S5.) As can be seen, a small subset of Markov models (about 0.07% of all sampled models) agrees with these new measurements within experimental uncertainty. Although the range of occupancies predicted by our Markov models is too broad to actually predict these experiments, they are consistent with these independent data.

**Figure 8:**
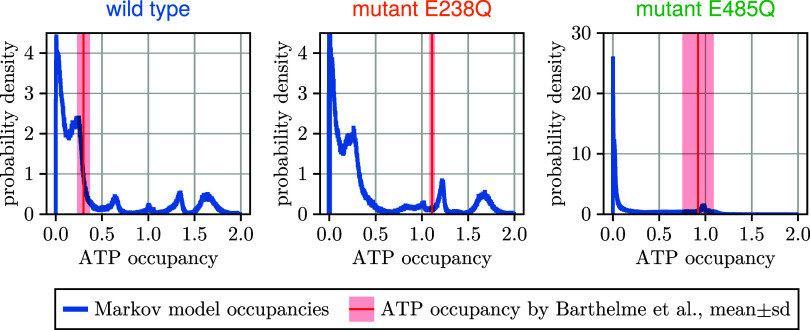
Ligand occupancies. Marginal posterior probability distribution of steady-state ATP occupancies for wild type (left), mutant E238Q (middle), and mutant E485Q (right); the measured ATP occupancies^7^ (red line) and experimental uncertainty (shaded, red) are shown for comparison.

The second occupancy measurement was performed by Nürenberg-Goloub et al.^26^ under nonequilibrium conditions. Specifically, a very large initial ABCE1 to ATP ratio (1:2) was used, which results in quickly decaying substrate concentrations even during the short 30 s reaction time of the experiment so that our steady-state assumption is no longer valid. We therefore resorted to numerical integration of the master equation (eq 4, see Methods) to model this experiment. Figure 9 compares the mean and standard deviation of the measured occupancy distributions by Nürenberg-Goloub et al.^26^ (pink crosses) with the occupancies calculated from the Markov models. Somewhat disappointingly, whereas our Markov model sample is consistent with the measured ATP occupancies, none of these reproduce the measured ADP occupancies. This discrepancy is caused by either too low hydrolysis rates in essentially all of our Markov models or too fast unbinding of the product ADP to reach the measured occupancies within 30 s, or both.

**Figure 9:**
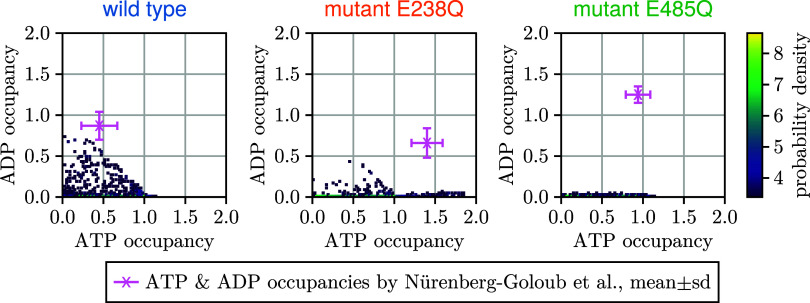
Ligand occupancies. Joint posterior probability distributions of single-turnover ATP and ADP occupancies are shown by color for wild type (left), mutant E238Q (middle), and mutant E485Q (right); the measured ADP and ATP occupancies^26^ (pink cross) and respective experimental uncertainties are shown for comparison.

Although this discrepancy does not invalidate our main conclusion that (and how) asymmetric kinetics can be achieved without direct allosteric interactions between the two binding sites, it does raise some doubts about whether our suggested mechanism is actually at work for the specific ABCE1 example at hand. To address this issue, we investigated the possible causes of this discrepancy in more detail.

First, we considered the possibility that Markov models without direct allosteric interaction that also agree with the above occupancy measurements do exist but contribute so little to the posterior (eq 2) that they were missed by our sampling protocol. To test this idea, we added both steady- and nonsteady-state occupancy data to the likelihood function and thus generated a new Bayes sample from all available experimental data. To identify those Markov models which agree best with experiments, we subsequently performed a first choice hill climbing search starting from the 20 sampled Markov models with the highest posterior probability. Figure 10 shows the nonsteady-state occupancies (top row, white circles) of the obtained Markov models. As can be seen, the resulting models are still inconsistent with the nonsteady-state occupancy data, suggesting that the discrepancy is not due to insufficient sampling. Instead, our model seems too simple to achieve agreement with all measurements within the experimental uncertainty. We consider this finding remarkable, given that a total of 17 free parameters seem insufficient to match 15 experimental data points.

**Figure 10:**
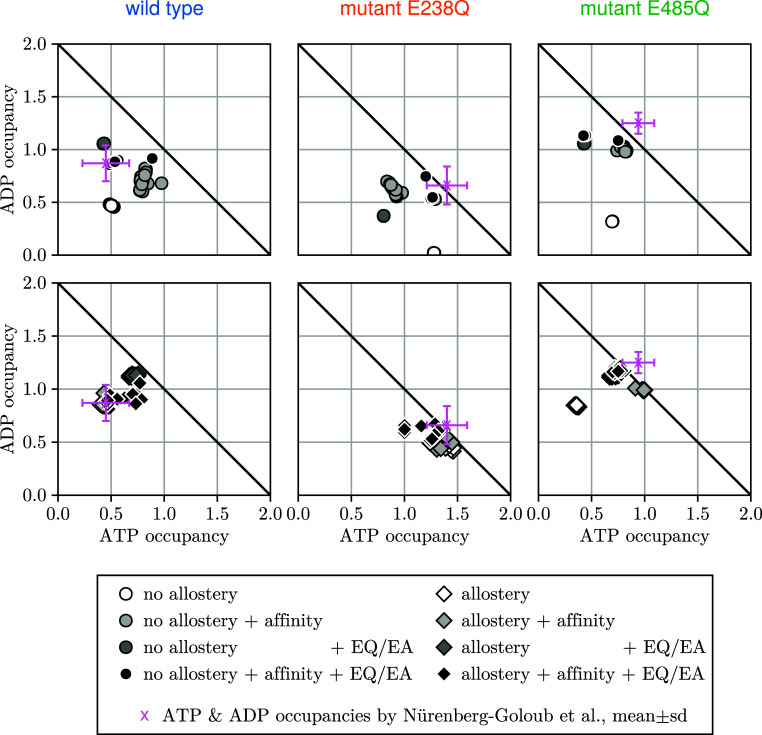
Comparison of ligand occupancies obtained for the different Markov classes discussed in the text. ATP and ADP occupancies without (top row, circles) and with (bottom row, diamonds) direct allosteric interaction for wild type (left), mutant E238Q (middle), and mutant E485Q (right). Different shades indicate Markov models that differ in how much the affinities are affected by the mutation (light gray), in how much the two different mutations used in the experiments (EQ vs EA) reduce the hydrolysis rate (dark gray), and both (black). In the lower left panel, the white diamond markers (Markov models with direct allosteric interaction) lay below the black diamond markers (Markov models with allostery and both adaptions). For each Markov model class, 20 symbols are shown, representing the 20 Markov models with highest posterior. The measured ADP and ATP occupancies^26^ and their experimental uncertainties are shown in pink. The black diagonal line indicates the maximum possible occupation.

Which of our model assumptions may cause this discrepancy? To answer this question, we considered the following three as the most likely and repeated the above analysis with all possible combinations of these assumptions dropped. First, we removed the constraint of our central assumption and included possible direct allosteric interactions between the binding sites within our Markov model class (see Figure S9). Notably, this modification nearly doubles the number of model parameters (32) compared to our original model without any direct allosteric interaction (17, see Figure 2). As can be seen in Figure 10 (bottom row, white diamonds), the agreement with the occupation data improves somewhat; however, despite the increased number of model parameters, marked discrepancies for the E485Q mutant occupancies persist. Notably, the mutant E485Q occupancies are rather similar to those of the wild-type, which may indicate that in order to fully explain all experimental data, further differences between the wild type and the two mutants—beyond inhibition of hydrolysis—need to be considered.

To test this idea, we extended our Markov model classes by including further potential effects of the mutations. In particular, we now assumed that the mutations reduce the ATP hydrolysis rate of each NBS by a different and unspecified factor between 10^–12^ and 1. Additionally, we allowed the mutations to affect the ATP and ADP affinities of each NBS separately. To this end, we further assumed the effect on the affinities to be mostly of electrostatic nature; hence, due to the negative charge of ADP and ATP, removal of the negatively charged glutamate acid should increase the affinity. Nevertheless, due to the absence of suitable affinity measurements, we also allowed small affinity decreases of up to 3 kT. Figure 10 compares the resulting occupancies for Markov model classes with and without any direct allosteric interaction (top row, light gray circles; bottom row, light gray diamonds). Indeed, even for the Markov models without direct allosteric interactions, much better agreement is seen now also for mutant E328Q; similar improvements are seen for models with direct allosteric interactions, here particularly for E485Q. Overall, these two generalizations of our simple model largely improved agreement with the occupancy measurements while maintaining agreement with all other measurements (Figure S10). Specifically, these modifications were required to reproduce the experimentally observed occupancy differences between all three species.

To address the remaining smaller discrepancies, we finally asked if these might be due to the fact that different mutations were used for the steady-state (glutamic acid to glutamine) versus the nonsteady-state (glutamic acid to alanine) experiments. To this end, we further extended all previous Markov model classes by allowing for two different sets of ATP hydrolysis reduction factors and affinity changes for the steady-state and nonsteady-state experiments, respectively, thereby increasing the total number of free parameters to 29 for the Markov models without any direct allosteric interaction and to 44 parameters for the Markov models including allostery (17 and 32 parameters for the wild-type transition rate coefficients plus 12 parameters for mutation effects, respectively). Despite such marked surplus of fit parameters relative to the 15 experimental values, still small discrepancies of $\frac{1}{2}\sigma$ or less remain.

Which of the eight considered Markov model classes best explains all measurements, taking into account their different numbers of parameters? To answer this question, one would ideally compare the total probabilities of the respective posteriors; however, due to the high dimensionalities of the search spaces, the complexity of the probability landscapes, and the computationally intensive calculation of single point posteriors, we found this calculation infeasible and thus resorted to the Bayesian information criterion^67,68^ (BIC), which “punishes” model complexity by *k* log (*n*), where *k* is the number of model parameters and *n* is the number of experimental data points. Figure 11 shows the BIC values for all eight Markov model classes. As can be seen, any model refinement relative to the prototypic original model (white circle) improves the BIC considerably. Notably, inclusion of direct allosteric interactions alone (white diamond) seems to be less effective than any of the other two generalizations, even considering their much larger number of free parameters. This finding suggests that direct allosteric interaction is not the key determinant for the observed kinetic asymmetry. Further support for this notion is provided by the fact that for the most general Markov model class (black diamonds), the achieved best agreement with the experiment is counterbalanced by its large number of parameters, such that a Markov model class without any direct allosteric interaction achieves the best trade-off and the lowest BIC.

**Figure 11:**
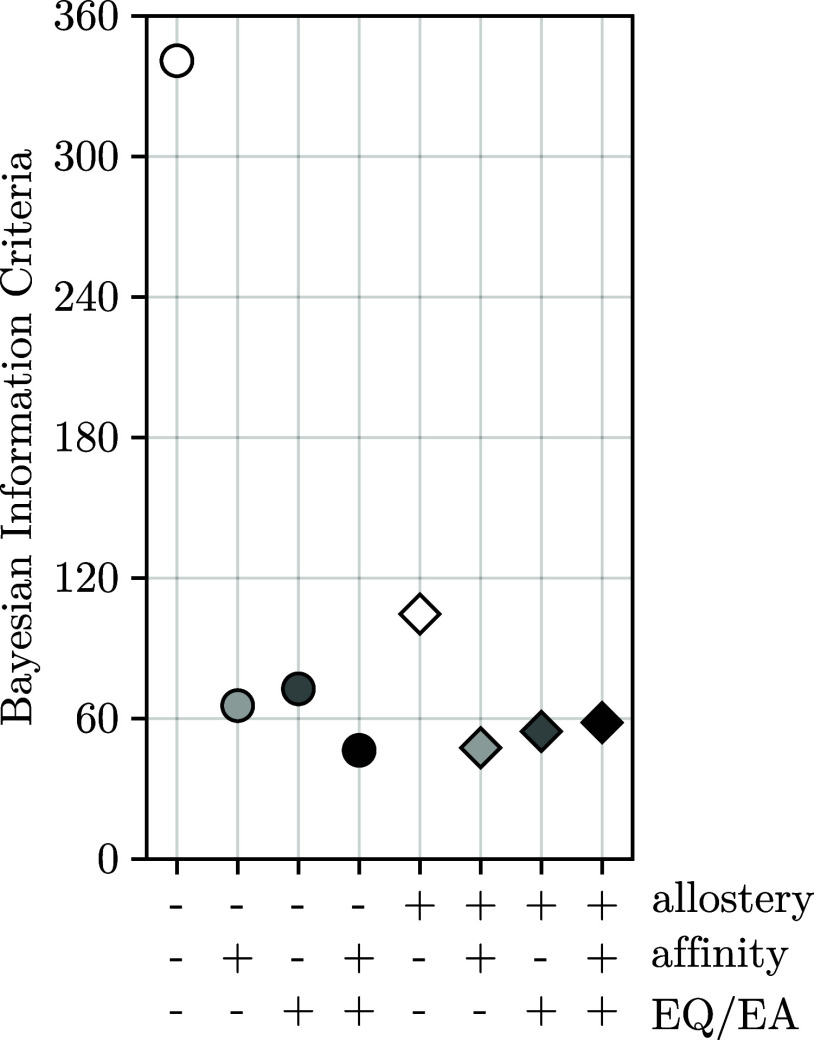
Markov model class comparison. The Bayesian information criterion is shown for the Markov model classes with (circles) and without (diamonds) direct allosteric interaction between NBSs. Different shades indicate the Markov model classes, which either include mutations affecting affinities and ATP hydrolysis rates differently for each mutant (light gray), include differences between EQ and EA mutations of the catalytic active glutamic acid in the Walker-B loop (dark gray), or include both (black).

## 3 Conclusion

The ATPase ABCE1 has two nearly identical NBSs, in terms of both sequence and structure, suggesting that each NBS contributes equally to the overall ATP turnover rate. Recently, two mutants have been studied^7^ to probe this near symmetry, each rendering one of the two NBSs ATP-hydrolysis defective. Only one of these mutants (E238Q) shows the expected 2-fold decrease of ATP turnover rate, whereas, quite unexpectedly, defectiveness of the other NBS (mutant E485Q) results in a staggering 10-fold increase. A straightforward explanation would be an allosteric communication in terms of a direct interaction between the two sites, e.g., that the presence of ATP or ADP in one site strongly affects the binding affinity or hydrolysis rate of the other. Because for the ABCE1 system at hand this explanation is rather unlikely due to the high level of symmetry, we here asked whether the observed striking kinetic asymmetry between the two mutants can be explained also without any such direct allosteric interaction.

To answer this question, we described the combined conformational and chemical kinetics of ABCE1 by Markov models which, by construction, are thermodynamically consistent but lack any direct communication between the two NBSs. As “minimal models”, our Markov models comprised 13 separate Markov states (each representing a combination of open and closed conformations with empty, ADP, and ATP occupation of each NBS) and physicochemically plausible transitions between these states. The respective transition rate coefficients were then determined via Bayesian inference, using the measured ATP hydrolysis kinetics. In particular, a large Bayesian posterior sample of Markov models with specific transition rate coefficients for each transition was generated, yielding a posterior probability to agree with the measured data for each of these models. This posterior served to identify a subset of all possible Markov models that agree with the observed ATP hydrolysis kinetics within experimental error, including the observed kinetic asymmetry. The fact that such a subset of Markov models exists demonstrates that indeed, a direct allosteric communication between the NBSs is not required to explain the observed kinetic asymmetry.

This is the main result of this study. Because, obviously, an enzyme with two fully independent NBSs cannot exhibit the observed hyperactivity, we conclude that it is caused by the concerted opening and closing motions of the two protein domains. This conformational dynamics provides some communication between the two NBSs—albeit very indirectly—and was previously not considered a suitable explanation for the observed asymmetry or any allostery in general.

Closer inspection of the identified Markov models provided mechanistic insight into how asymmetric ATP hydrolysis kinetics can be achieved. The most striking property, shared by almost one-fifth of all Markov models that quantitatively agree with experiment and thus reproduce the observed asymmetry, is that in the wild type two separate reaction cycles contribute to the overall ATP turnover rate. Unexpectedly, both involve hydrolysis in NBSI, such that by far most of the ATP hydrolysis occurs within NBSI. While both cycles contribute about equally to the overall hydrolysis turnover rate, their population differs drastically by two to 3 orders of magnitude. In fact, the open ABCE1 conformation with ATP-loaded NBSI and ADP-loaded NBSII is by far the most populated Markov state. This population asymmetry is compensated by a corresponding rate coefficient asymmetry for the two cycles, resulting in similar fluxes, each contributing ca. 50% to the overall hydrolysis rate. Crucially, ATP hydrolysis within NBSII connects these two cycles, thus creating a “drainage” that creates this population asymmetry by steadily depleting the sparsely populated cycle in favor of the highly populated one. By blocking this drainage in the mutant E485Q, a fraction of the dominant population shifts toward the low-populated cycle, thus enhancing its contribution to the overall hydrolysis rate from 50% to about 20-fold.

Using steady-state and new nonequilibrium measurements, we performed two rounds of cross-validation aiming to check if our Markov model accurately describes the ATP hydrolysis kinetics of the ABCE1 wild type and the two mutants that knock out one of the active sites each. While the first cross-validation showed consistency with the new data, the second did not.

As the three most likely possible reasons for this remaining discrepancy, we considered (1) the presence of direct allosteric interactions, (2) additional effects of the mutation onto ATP and ADP affinities, and (3) differences between the mutations used in the experiments and performed full Bayesian samplings on all combinations of these three. As one should expect, all extensions of our initial prototypic Markov model class resulted in somewhat improved agreement with all available experimental data. Strikingly, experimental details such as (2) and (3) seemed to explain the remaining discrepancies better than the presence of direct allosteric interactions, which suggests that the latter are less likely to dominate the observed kinetic discrepancy. Aiming at extracting the main mechanism, we therefore consider it appropriate to base our analysis largely on the simplest Markov model. This notion is supported by the unexpected finding that despite the largely increasingly underdetermined character of all eight models considered, full agreement with all experimental data was not achieved even for the most complex model. We speculate that further structural differentiations, such as inclusion of a half-open conformation or further details of the experiments that have so far not been included within our physical models, may be relevant. At present, given the limited experimental data and the complexity of the actual protein dynamics, we think that our initial prototypic Markov model provides a good balance.

Obviously, future experiments will be necessary to further refine the specific mechanism of ABCE1 and other proteins. Recent advances using nanoaperture optical tweezers,^69^ which enable time-resolved^70^ and label-free observations of conformational^71^ and binding dynamics^72^ at the single-molecule level, provide a quite promising complementary approach.

In summary, and independent of the above experimental and model details and refinements specific to ABCE1, this study revealed an unexpectedly complex behavior of Markov models, which can provide a thermodynamically consistent description of equilibrium, steady-state, and nonequilibrium behavior of proteins that achieve their function through a tight interaction between conformational motions and chemical reactions.

Even for systems as simple as the ATPase ABCE1, which comprised only two active sites and only two conformers, the resulting complexity of a 13-state minimal Markov model gives rise—and can explain—quite counterintuitive behavior. Specifically, using this type of Markov model, our study suggests a mechanism by which striking ATP hydrolysis kinetics asymmetry of enzymes such as ABCE1 does not require any direct allosteric interactions between the two NBSs (nor does it rule these out, though); rather, we have shown that this asymmetry can be explained solely in terms of concerted opening/closing conformational motions of this dimer. Notably, the 10-fold enhanced overall ATP hydrolysis rate upon removal of the “drainage” represents a striking molecular example of the so-called Braess’ paradox,^73^ which in its original formulation states that removal of a road can enhance traffic throughput.

## 4 Methods

### 4.1 Markov Models

We use time-continuous Markov models to describe ABCE1 as a set of discrete states with memory-free transitions between them (Figure 2). The probability (per second) of such a transition from state *i* to state *j* is determined by its respective transition rate $\left\{k_{ji}\right\}\in R_{\geq 0}$ The probability of ABCE1 to be in a state *i* at a given time *t* is given by *X*_*i*_(*t*), and, given an initial distribution ***x*** (0), the time evolution of the probabilities ***x*** (*t*) is determined by the master equation

4 ẋ=Q·x(t)

a system whose transition rate matrix ***Q*** contains the rate coefficients *k*_*ji*_ and diagonal elements $k_{jj}=-\sum_{i=1,i\neq j}^{N}k_{ji}$ , where *N* is the number of states. Note that *k*_*ji*_ are referred to as transition rates in the context of Markov models, but hereafter, we refer to them as transition rate coefficients *k*_*ji*_ to be consistent with chemical terminology. For irreducible and aperiodic Markov models, the probabilities ***x*** (*t*) converge toward unique stationary or steady-state probabilities **π** for *t* → ∞, such that

5 0=Q·π

The state probabilities **π** were calculated by solving the system of linear equations, eq 5, without integration of the master equaiton, eq 4.^74^ Given ***x*** (*t*), the net flux of a transition, i.e., how much probability (per second) is transferred between states *i* and *j*, is *k*_*ji*_ *x*_*i*_ – *k*_*ij*_ *x*_*j*_, where the signs are chosen such that the net flux is positive if ATP is hydrolyzed.

The linearity of eqs 4 and 5 implies that Markov models are inherently limited to describing first-order reactions. In order to also include the higher-order reactions of ATP-, ADP-, and P_*i*_-binding within the Markovian framework, and following the steady-state assumption,^75^ we assume that the change of these concentrations is negligible at the time scale of the hydrolysis cycle and include these concentrations as constant factors of the respective transition rate coefficients in matrix ***Q***, for example, the transition rate coefficient for ATP-binding was chosen as *k*_J_ [ATP].

As discussed in the Results section, we further described ADP-binding and P_*i*_-binding by a single rate-limiting transition with effective transition rate coefficients. To ensure that the free energy difference along each closed cycle including a net ATP hydrolysis still equals the Gibbs free energy of ATP hydrolysis ( $\Delta G_{ATP⇌ADP+P_{i}}^{{}^{\circ}}=$ −29.288 kJ mol^–1^);^76^ for thermodynamic consistency, the transition rate coefficient for ADP + P_*i*_-binding was chosen as *k*_H_ [ADP]­[P_*i*_].

As also discussed in the Results section, we parameterized our Markov model classes without direct allosteric interaction by 17 free energy parameters, of which 7 were free energy differences (free energy difference associated with the conformational change Δ*G*_open→closed_, two free energy differences between ATP and ADP bound in the closed state Δ*G*_ATP→ADP, NBSI_ and Δ*G*_ATP→ADP, NBSII_, and four free binding energy differences Δ*G*_ATP binding, NBSI_, Δ*G*_ADP unbinding, NBSI_, Δ*G*_ATP binding, NBSII_, and Δ*G*_ADP unbinding, NBSII_) and 10 were forward free energy barriers (Δ*G*_A_^‡^, Δ*G*_B_^‡^, ..., Δ*G*_J_^‡^ one for each transition A, B, ..., J).

The Markov model classes with direct allosteric interaction are parameterized by 32 free energy parameters, of which 12 were free energies (one per Markov state with the free energy of one Markov state set to zero) and 20 were forward free energy barriers (Δ*G*_A_^‡^, Δ*G*_B_^‡^,···, Δ*G*_T_^‡^ one for each transition A, B,···T; cf. Figure S9).

For the Markov model classes without an effect of the mutation on the affinities, the mutations result in a 100-fold reduction of the ATP hydrolysis transition rates coefficients (*k*_B_ and *k*_C_ for Markov model classes without as well as *k*_B_, *k*_C_, *k*_E_, and *k*_F_ for Markov model classes with direct allosteric interaction) and the corresponding ATP synthesis transition rate coefficients. In contrast, for Markov model classes including an effect of the mutation on the ATP and ADP affinities, this reduction factor is different for each NBS and is added as a parameter to the Bayesian inference with a prior that is log-uniformly distributed within the interval [10^–12^, 1]. To allow for different affinities, four additional factors are defined (one per nucleotide and per binding site) and also added as a parameter with a uniform prior between −10 kT and 3 kT. These affinity factors scale all transition rate coefficients that go from states with the respective nucleotide in the respective binding site to states with a different occupation, e.g., going from ATP to ADP or apo. This assumes that mostly unbinding rates are affecting by the mutation.

For each experiment, the transition rate coefficients were scaled to the experimental temperature in accordance with transition state theory.^60^

A working code example of the Markov models is available at https://gitlab.gwdg.de/mschaef6/abce1_mm.

### 4.2 Description of Steady-State Experiments

The measurements of limiting ATP turnover rate *k*_cat_, Michaelis–Menten constant *K*_M_, and ATP occupancy were assumed to be performed under steady-state conditions.^7^ Therefore, in order to calculate the posterior (eq 2), these observables had to be calculated from eq 5.

Based on the steady-state solution **π** of eq 5, the ATP turnover rate was calculated as the sum of the net fluxes of all ADP-unbinding transitions for ATP concentrations between 10^–9^ and 10^2^ in logarithmic steps of 10.

The saturating ATP turnover rate *k*_vmax_ was calculated as the ATP turnover rate at a concentration of 1 × 10^2^ mol/L ATP.

The Michaelis–Menten constant was calculated from the ATP turnover rates determined by linear interpolation from two concentrations for which the turnover rates were close to *k*_vmax_/2. These two concentrations were determined from the two closest concentrations found for the above logarithmic grid by calculating ATP turnover rates for 18 additional logarithmically spaced concentrations between those and again selecting those two closest to *k*_vmax_/2. This approximate calculation was chosen over more accurate ones because of the need for high computational efficiency for the extensive Bayes-sampling.

To construct transition rate matrix ***Q*** of eq 5, ADP and P_*i*_ concentrations had to be chosen. The initial experimental ADP and P_*i*_ concentrations were zero;^7^ however, due to ATP hydrolysis by ABCE1, these concentrations were on the order of the ABCE1 concentration during the experiment. To account for this increase, we selected the ADP and P_*i*_ concentrations to be 100-fold the median experimental ABCE1 concentration (5 × 10^–6^ mol L^–1^),^7^ assuming multiple completed ATP hydrolysis cycles of ABCE1. We opted for this more rudimentary estimate, assuming that deviations from these ADP and P_*i*_ concentrations would exert a negligible influence on the calculated observables, which is consistent with the finding that ADP-/P_*i*_-binding is not identified as the rate-limiting step in any Markov model.

All ATP/ADP occupancies were calculated from the steady-state population via $\sum_{i}^{N}n_{i}\pi_{i}$ , where *n*_*i*_ are the respective ATP/ADP occupation numbers {0,1,2}. To estimate the ligand concentrations at the time of the measurement, we used the integrated Michaelis–Menten kinetics, i.e., assuming a steady-state at each point of time, and used the mean of concentrations between 4 and 5 min.

### 4.3 Description of Nonsteady-State Experiments

The ATP and ADP occupancy measurements by Nürenberg-Goloub et al. were performed under single-turnover conditions with a 2:1 ratio of ATP to ABCE1 ^26^ and, therefore, the above steady-state assumption does not hold. We therefore resorted to numerical integration of eq 4, which in this case become third order, with initial values [ABCE1] = 0.3 × 10^–6^ mol/L, [ATP] = 0.6 × 10^–6^ mol/L, and [ADP] = [P_*i*_] = 0 mol/L. The numerical integration was performed using the Rodas4P^77^ algorithm with a maximal time step of 0.1 s, relative tolerance 10^–6^, and 10^6^ maximum steps, for a duration of 30 s, to obtain ***x*** (*t* =30 s). The initial conditions assumed were that all ABCE1 is in an open conformation and that both NBSs are empty.

### 4.4 Bayesian Approach

For the calculation of the Bayesian posterior, we used a uniform prior for all 17 free energy parameters with boundaries chosen separately for chemical reactions, conformational transitions, and un/binding events following physical plausibility (summarized in Table 1). To facilitate implementation, these boundaries were defined in terms of transition rate coefficients rather than free energies. For thermodynamic consistency, only those Markov models that satisfy detailed balance were considered.

**Table 1:** Summary of Prior Boundaries Used for Bayes Sampling^a^

type of transition	lower bound	upper bound
ligand binding	10^–6^ L^2^ mol^–2^s^–1^	10^7^ L^2^ mol^–2^s^–1^
ligand unbinding	10^–6^ L mol^–1^s^–1^	10^7^ L mol^–1^s^–1^
chemical reaction	10^–6^ L mol^–1^s^–1^	10^12^ L mol^–1^s^–1^
conformational transition	10^–6^ L mol^–1^s^–1^	10^6^ L mol^–1^s^–1^

a The upper limit for chemical reactions was assumed to be faster than that of binding/unbinding events or conformational transitions.

For the Bayes-sampling (eq 2), we used a replica-exchange Markov-chain Monte Carlo algorithm with a slice sampler for local exploration with 8 chains of 32768 Markov models each using the Julia package Pigeons.^61^ To provide a quantitative assessment of convergence, Table S2 lists *R̂*-values^78^ of each chain and free energy parameter, in addition to the qualitative discussion of convergence in the Results section. *R̂*-values close to 1 indicate sufficient convergence of most chains with slightly worse convergence of individual chains, for example, chain 2 of Δ*G*_B_^‡^ with *R̂* = 1.05935.

For the Bayes-sampling of the Markov model classes considering direct allosteric interaction, additional effects of the mutation onto ATP and ADP affinities, and differences between the mutations used in the experiments, the same setup as above was used except that each chain was run for 24 h instead of a fixed chain length. Then, for each Markov model class, the 20 Markov models with highest posterior were selected as starting point for a 2 h long stochastic hill climbing search, where new Markov models are randomly generated until a Markov model with higher posterior is found (first choice hill climbing).^79^

### 4.5 Rate-Limiting Step

To calculate the rate-limiting step, we used back-propagation to calculate the derivative of the overall ATP turnover rate with respect to each unique transition rate (20) of a Markov model.

## Supplementary Material



## Notes

Definition of transition rate coefficients; Bayesian sampling convergence assessments; additional data analyses of sampled Markov models; and Markov model scheme with direct allosteric interactions (PDF)

## §

Department of Chemistry, Wesleyan University, 52 Lawn Ave, Middletown, Connecticut 06459, United States
