# Supplementary material for: Braess’ Paradox in Enzyme Kinetics: Asymmetry from Population Balance without Direct Cooperativity
Source: J Chem Theory Comput. 2026 Feb 4;22(4):1982–99. doi: 10.1021/acs.jctc.5c01269 (PMC12937102; doi:10.1021/acs.jctc.5c01269)
Supplement: Supplementary file 1 [file ct5c01269_si_001.pdf]

Supporting Information: Braess' Paradox in Enzyme Kinetics: Asymmetry from Population Balance without Direct Cooperativity

Malte Schäffner,† Colin A. Smith,‡ Robert Tampé,¶ and Helmut Grubmüller*,†

†*Theoretical and Computational Biophysics, Max Planck Institute for Multidisciplinary Sciences, Am Fassberg 11, 37077 Göttingen, Germany*

‡*with present address: Department of Chemistry, Wesleyan University, 52 Lawn Ave, Middletown, Connecticut 06459, United States*

¶*Institute of Biochemistry, Biocenter, Goethe University Frankfurt, Max-von-Laue-Str. 9, 60438 Frankfurt am Main, Germany*

E-mail: hgrubmu@gwdg.de

Table S1: Definition of transition rate coefficients in Figure 2. Listed are Markov transitions (left column), which describe either conformational transitions, binding reactions, or chemical reactions (right column). Occupations are indicated for NBSI/NBSII.

transition rate coefficients		Markov transitions	
forward	backward	forward	backward
k_A	k_{-A}	open → closed, ATP/ATP	closed → open, ATP/ATP
k_B	k_{-B}	ATP → ADP, NBSI	ADP → ATP, NBSI
k_C	k_{-C}	ATP → ADP, NBSII	ADP → ATP, NBSII
k_D	k_{-D}	closed → open, ADP/ATP	open → closed, ADP/ATP
k_E	k_{-E}	closed → open, ATP/ADP	open → closed, ATP/ADP
k_F	k_{-F}	closed → open, ADP/ADP	open → closed, ADP/ADP
k_G	k_{-G}	ADP unbinding, NBSI	ADP binding, NBSI
k_H	k_{-H}	ADP unbinding, NBSII	ADP binding, NBSII
k_I	k_{-I}	ATP binding, NBSI	ATP unbinding, NBSI
k_J	k_{-J}	ATP binding, NBSII	ATP unbinding, NBSII

Table S2: Quantitative per chain Bayes-sampling convergence assessment. Listed are $\hat{R}$-values[61] per chain and per free energy parameter.

parameter	chain 1	chain 2	chain 3	chain 4	chain 5	chain 6	chain 7	chain 8
$\Delta G^{\ddagger}_{A}$	1.00747	1.00166	1.03971	1.00049	1.00634	1.00095	1.00589	1.00019
$\Delta G^{\ddagger}_{B}$	1.00175	1.05935	1.00743	1.00177	1.00017	1.01509	1.01703	1.00976
$\Delta G^{\ddagger}_{C}$	1.00135	1.00011	1.02143	1.0034	1.01301	1.01833	1.00405	1.01165
$\Delta G^{\ddagger}_{D}$	1.00073	1.00082	1.00522	1.00279	1.00028	1.00254	1.00032	1.02118
$\Delta G^{\ddagger}_{E}$	1.00539	1.00084	1.00183	1.0012	1.01355	1.01616	1.00363	1.00088
$\Delta G^{\ddagger}_{F}$	1.00219	1.03133	1.00286	1.00016	1.00281	1.00313	1.0065	1.00103
$\Delta G^{\ddagger}_{G}$	1.01779	1.00744	1.00355	1.00139	1.01159	1.04002	1.00034	1.03073
$\Delta G^{\ddagger}_{H}$	1.00096	1.00381	1.00149	1.0012	1.00018	1.00131	1.00431	1.02721
$\Delta G^{\ddagger}_{I}$	1.00005	1.00221	1.00047	1.00053	1.00025	1.00273	1.00004	1.00168
$\Delta G^{\ddagger}_{J}$	1.00872	1.00485	1.00949	1.00038	1.00822	1.00798	1.00082	1.00445
$\Delta G_{\text{ATP binding, NBSI}}$	1.01087	1.01359	1.00862	1.0001	1.01316	1.02756	1.01616	1.01208
$\Delta G_{\text{ADP unbinding, NBSI}}$	1.00381	1.00191	1.00123	1.00028	1.00568	1.00789	1.00424	1.00298
$\Delta G_{\text{ATP binding, NBSII}}$	1.00542	1.0072	1.01108	1.00041	1.00749	1.00218	1.00056	1.00089
$\Delta G_{\text{ADP unbinding, NBSII}}$	1.00615	1.00132	1.01301	1.00063	1.00114	1.00431	1.00867	1.00044
$\Delta G_{\text{open}\rightarrow\text{closed}}$	1.00065	1.01764	1.01318	1.00089	1.00436	1.00556	1.00448	1.00916
$\Delta G_{\text{ATP}\rightarrow\text{ADP, NBSI}}$	1.00004	1.01081	1.00268	1.006	1.00248	1.00097	1.00358	1.02105
$\Delta G_{\text{ATP}\rightarrow\text{ADP, NBSII}}$	1.00269	1.0014	1.01869	1.00165	1.00112	1.01113	1.00886	1.00224

Figure S1: Bayes sampling convergence assessment. Shown are sampled posterior probability densities of barrier heights $\Delta G^{\ddagger}$ and free energy differences ΔG for all transitions between Markov states. Histograms for 8 individual Markov Chains (thin black lines) as well as for all 8 chains combined (thick red lines) are shown. The inset (bottom right) lists $\hat{R}$-values for quantitative assessment of convergence.

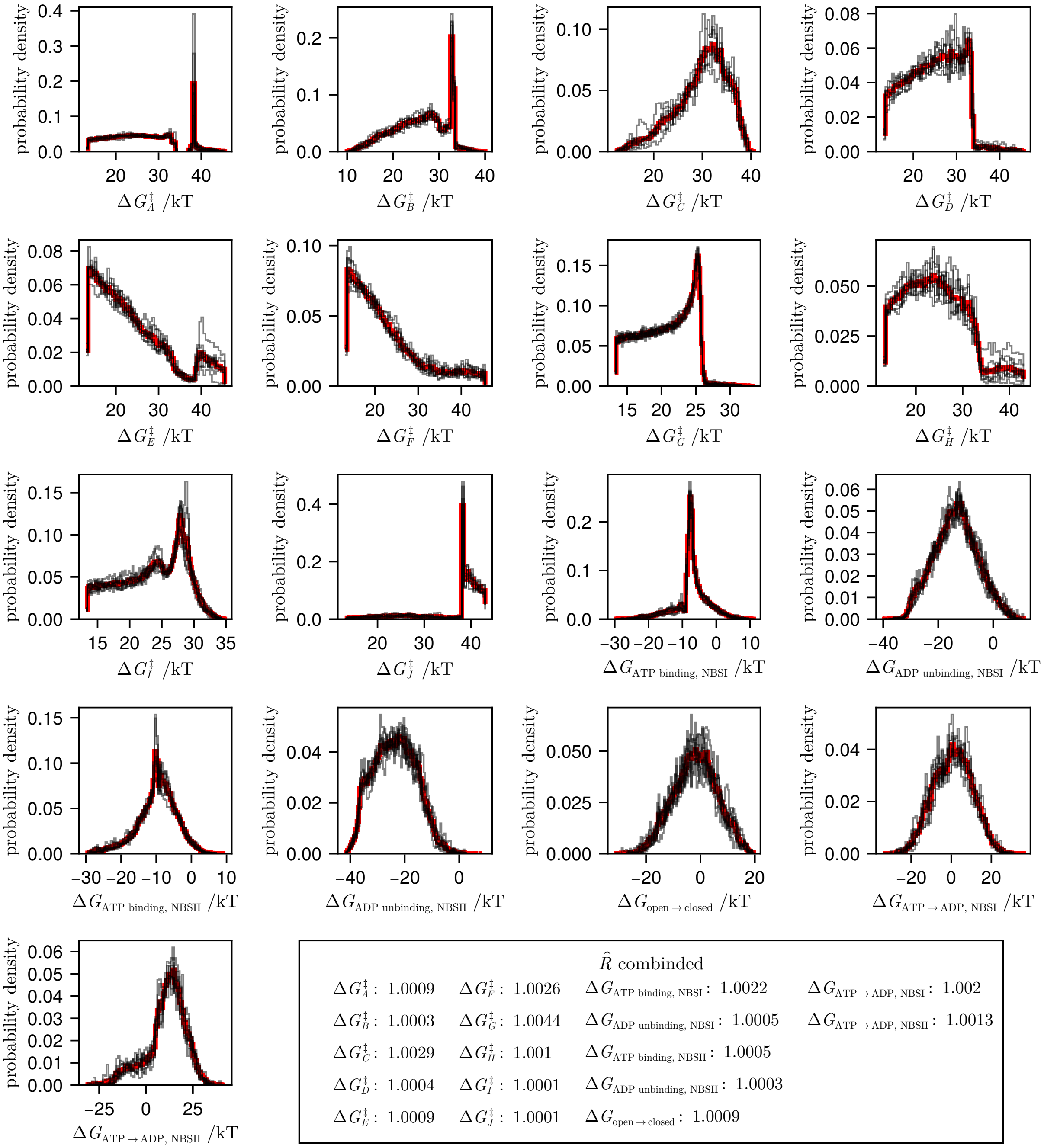

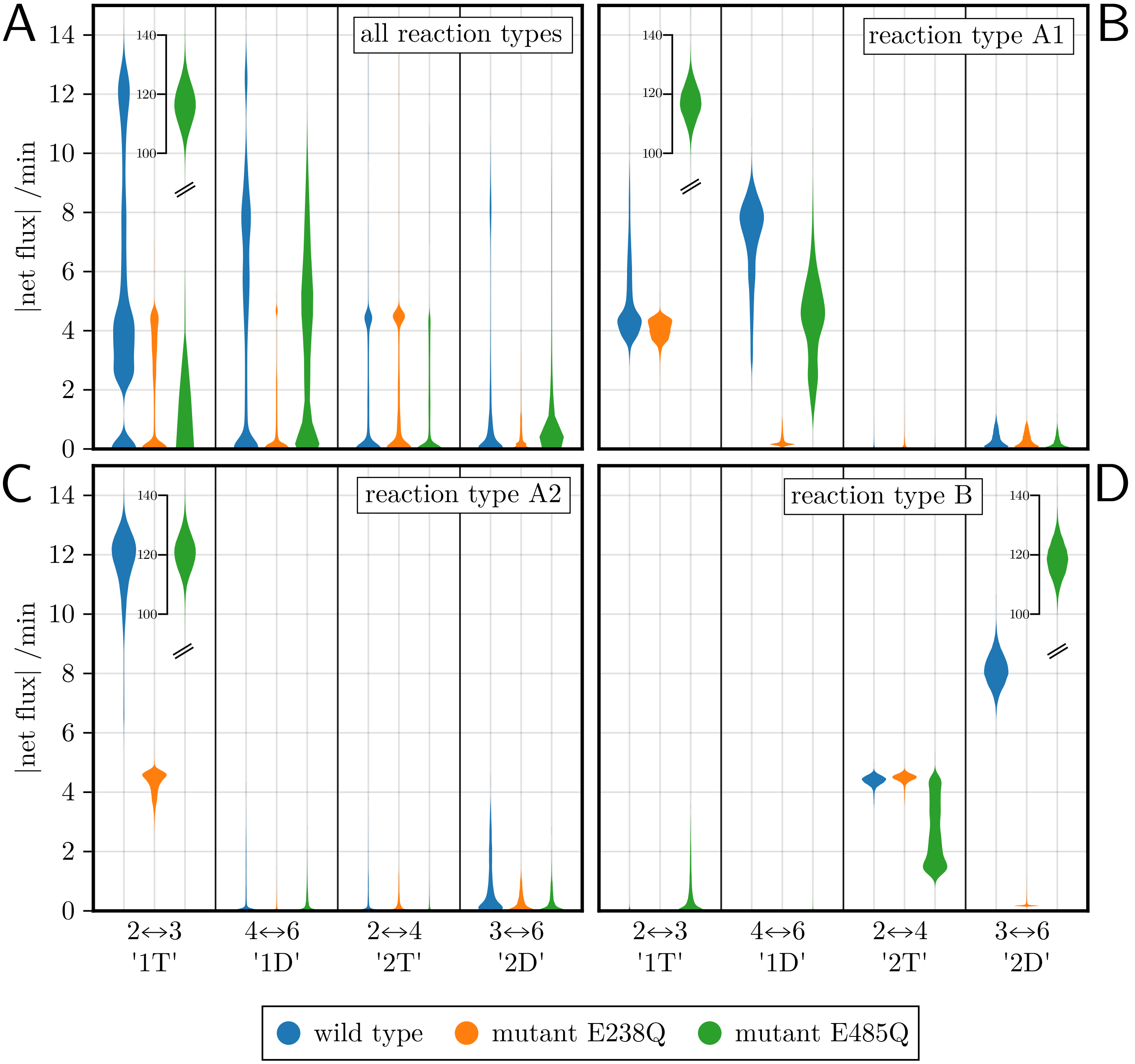

Figure S2: Net flux distributions, separated by ATP hydrolysis transitions. Shown are smoothed histograms of absolute net fluxes within Markov models for (A) all reaction types or (B-D) one of the three most probable reaction types (cf. Figure 4) for wild type and mutants (color). Annotations $i \leftrightarrow j$ indicate net fluxes between state i and j, which are dominated by net fluxes through reaction cycles '1T', '1D', '2T', and '2D' (cf. Figure 4). Note the discontinuities in the axis to accommodate the high net fluxes observed in the E485Q mutant.

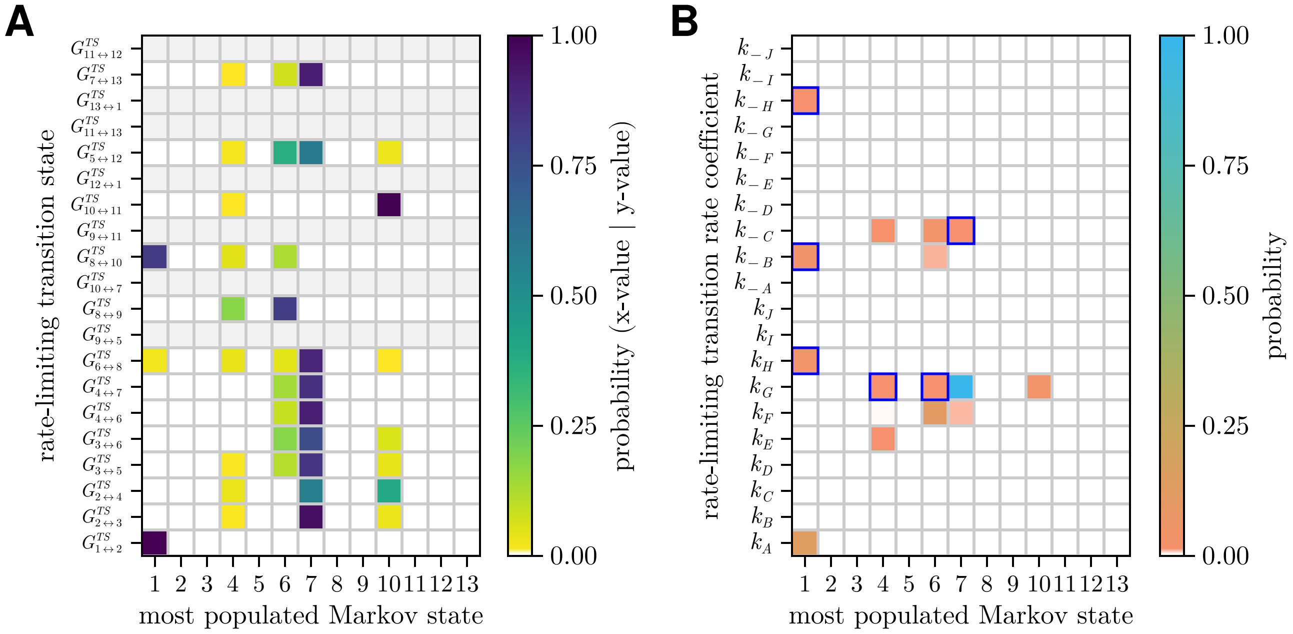

Figure S3: Comparison between most populated wild-type Markov states and kinetic determinants. (A) Shown are the conditional probabilities (color) of the most populated Markov state given each transition state to be rate-limiting. $G^{TS}_{i \leftrightarrow j}$ is the free energy of the transition state between state i and j. For transition states or transition rate coefficients that were not rate-limiting in any Markov model, the rows are empty (gray). (B) Shown are the probabilities of each combination of rate-limiting transition rate coefficient and most populated Markov state. Blue rectangles indicate combinations where the rate-limiting transition state does not belong to an outgoing transition of the most populated Markov state.

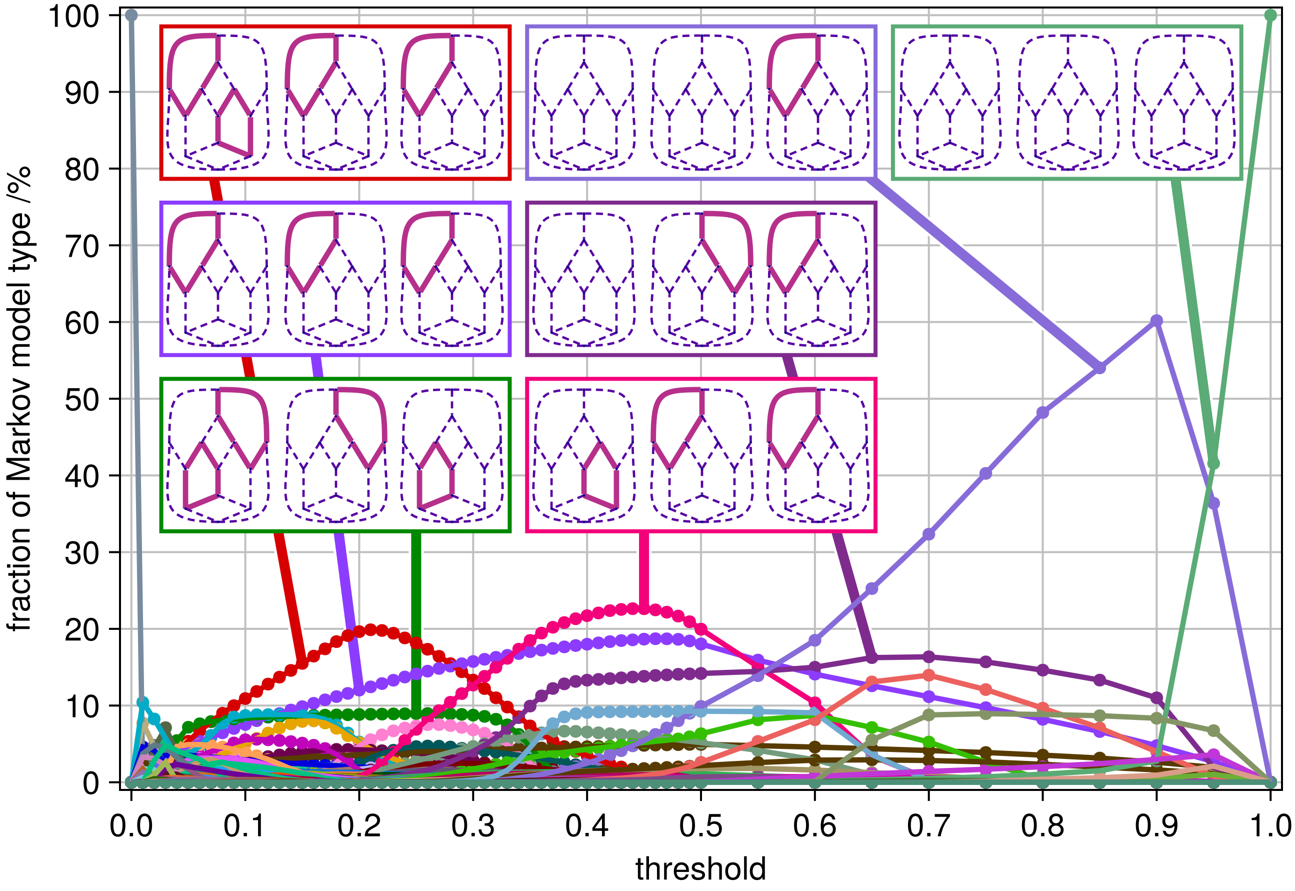

Figure S4: Dominant reaction types vary with chosen threshold. Shown are the posterior probabilities of the most prevalent reaction types (colored lines) for threshold values between 0 and 1. The insets indicate the respective dominant cycles as in Figure 4.

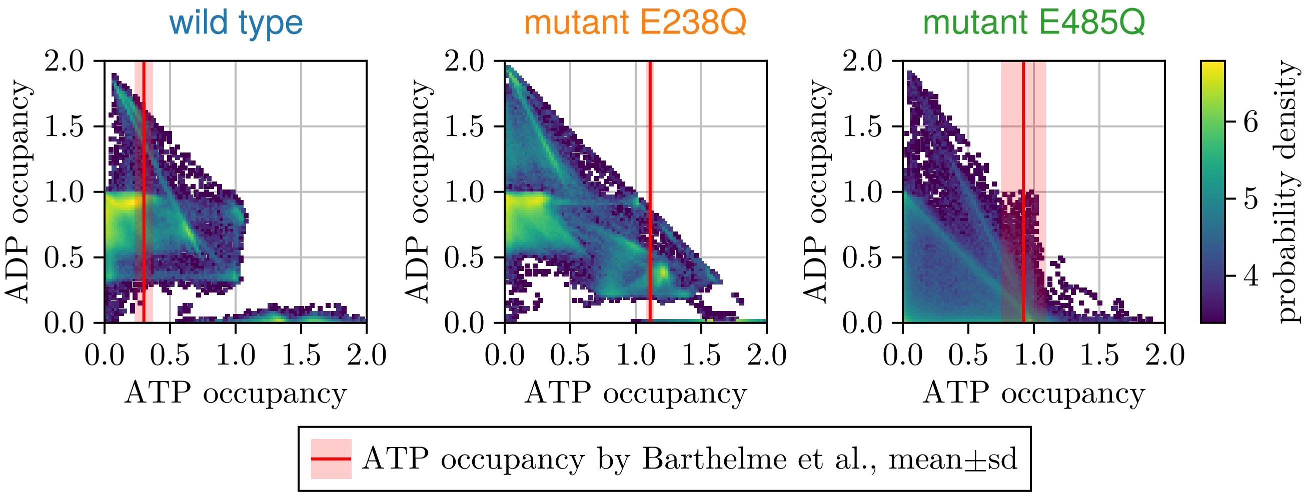

Figure S5: Ligand occupancies. Joint posterior probability distributions of steady-state ATP and ADP occupancies are shown by color for wild type (left), mutant E238Q (middle) and mutant E485Q (right); the measured ATP occupancies[7] (red line) and experimental uncertainty (shaded, red) are shown for comparison.

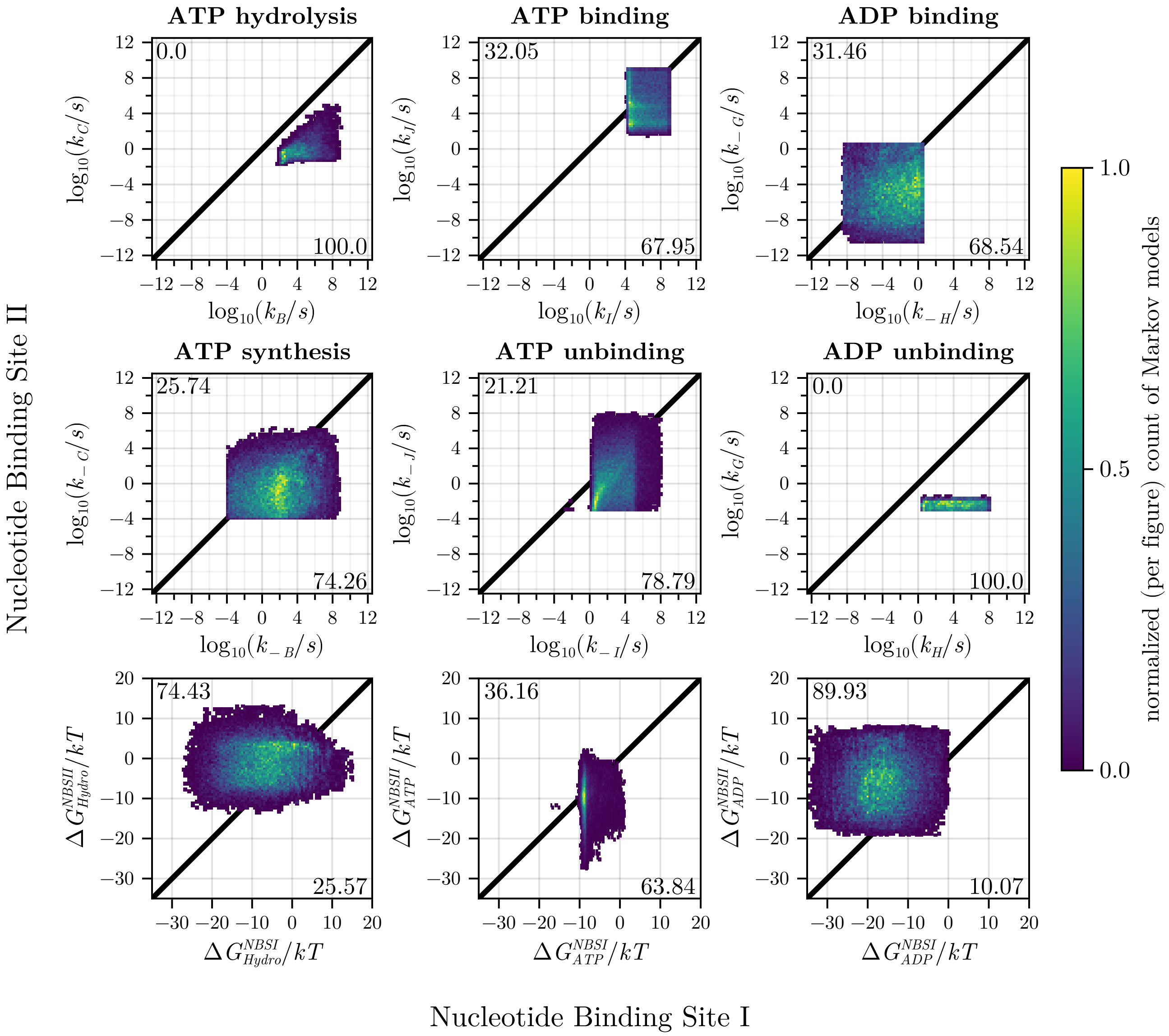

Figure S6: Comparison of nucleotide binding sites. Shown are 2d-histograms of forward and backward transition rate coefficients as well as free energy differences (rows, top to bottom) of ATP/ADP conversion, ATP exchange, and ADP exchange (columns, left to right) of Markov models assigned to the most probable reaction type A1 (cf. Figure 4). Numbers in each bottom right and top left corner indicate the percentage of Markov models for which either NBSI or NBSII has faster kinetics or a larger free energy difference, respectively.

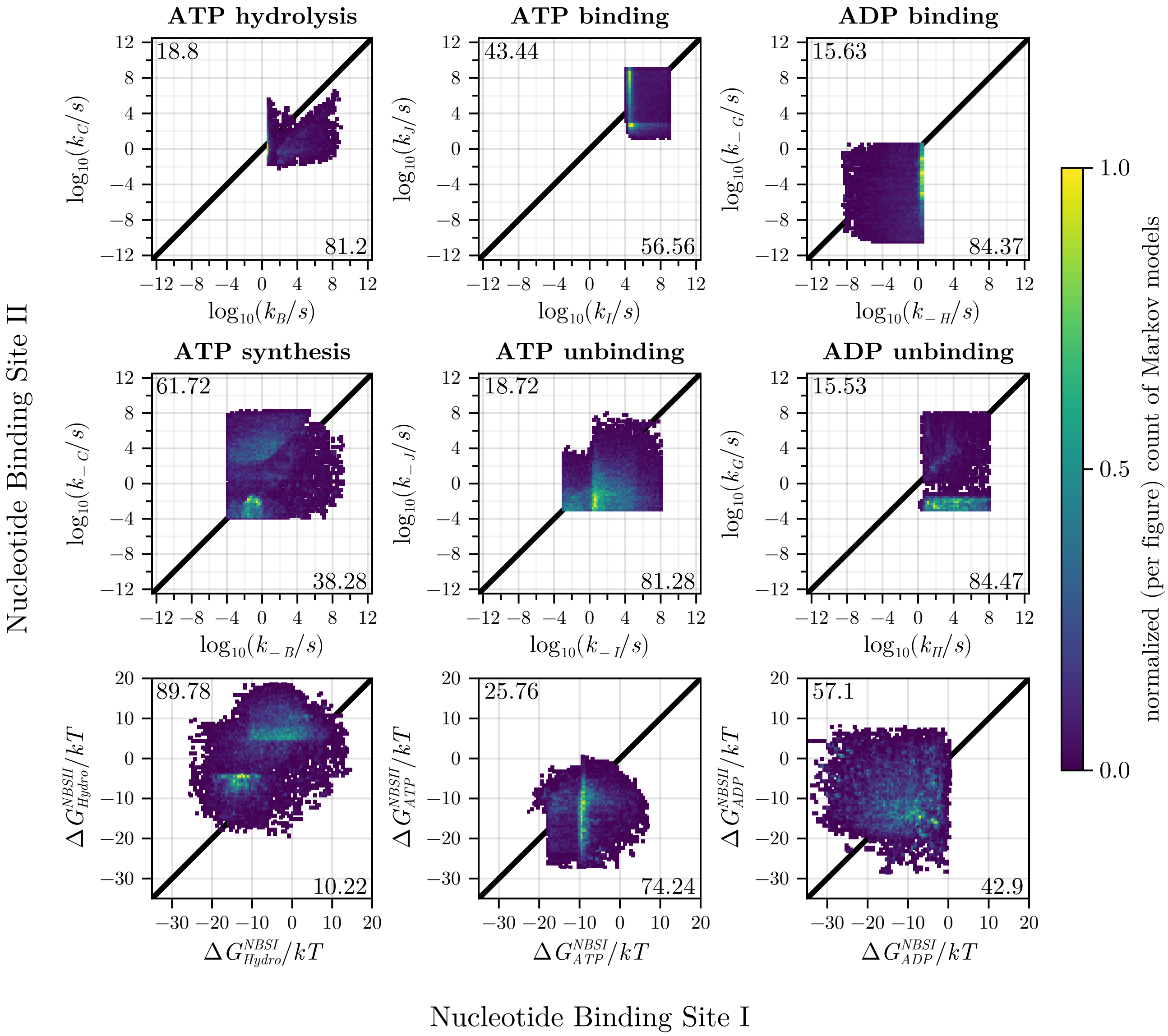

Figure S7: Comparison of nucleotide binding sites. Shown are 2d-histograms of forward and backward transition rate coefficients as well as free energy differences (rows, top to bottom) of ATP/ADP conversion, ATP exchange, and ADP exchange (columns, left to right) of Markov models assigned to the most probable reaction type A2 (cf. Figure 4). Numbers in each bottom right and top left corner indicate the percentage of Markov models for which either NBSI or NBSII has faster kinetics or a larger free energy difference, respectively.

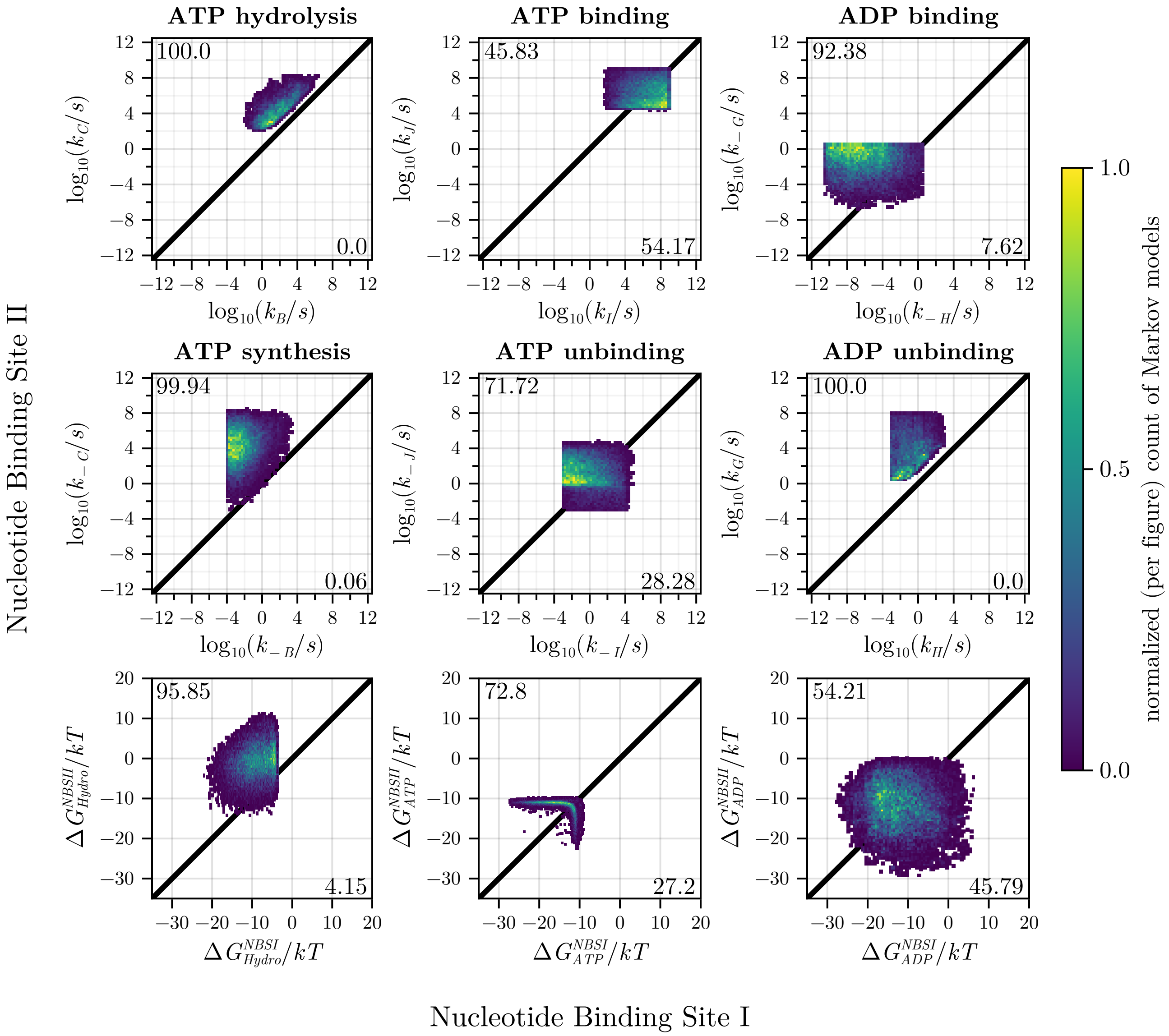

Figure S8: Comparison of nucleotide binding sites. Shown are 2d-histograms of forward and backward transition rate coefficients as well as free energy differences (rows, top to bottom) of ATP/ADP conversion, ATP exchange, and ADP exchange (columns, left to right) of Markov models assigned to the most probable reaction type B (cf. Figure 4). Numbers in each bottom right and top left corner indicate the percentage of Markov models for which either NBSI or NBSII has faster kinetics or a larger free energy difference, respectively.

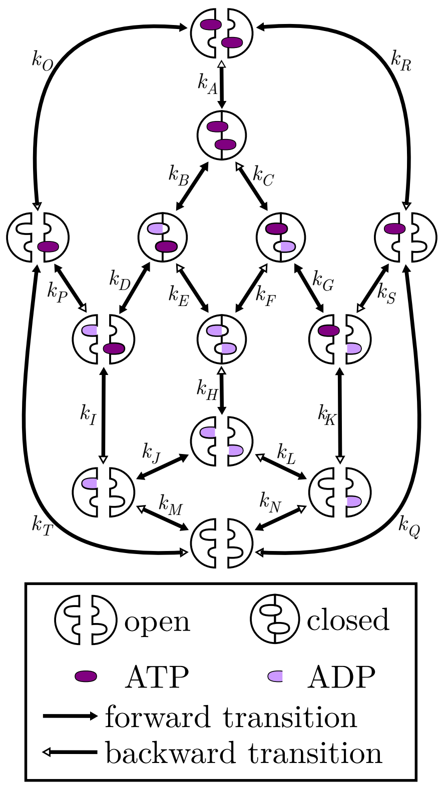

Figure S9: Graph representations of ABCE1 Markov model class with direct allosteric interaction. Each ABCE1 symbol represents one Markov state, which are connected by conformational or chemical transitions (arrows) with indicated transition rate coefficients k_i; due to direct allosteric interaction between the nucleotide binding sites all transitions have unique transition rate coefficients (cf. Figure 2). All transitions are reversible; for clarity only the forward rates (solid arrow heads) are annotated. Filled dark purple ovals denote ATP; partially filled light purple ovals denote ADP, indicating the one missing phosphate; for clarity inorganic phosphate is not shown.

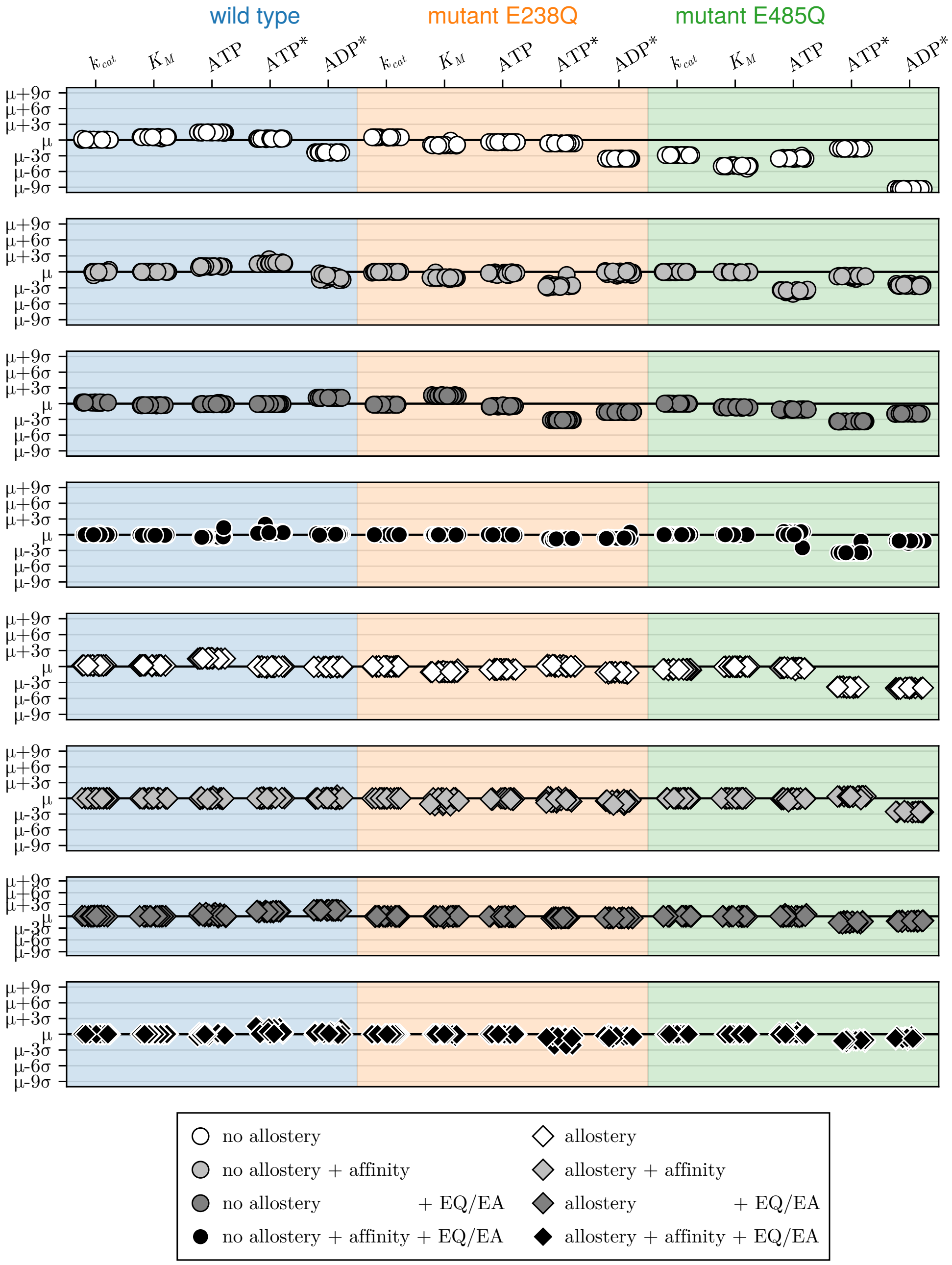

Figure S10: Agreement with experiment of different Markov model variants. The values of ATP turnover rate, Michaelis-Menten constant, steady state ATP occupancy (ATP), non-steady state ATP (ATP*) and ADP (ADP*) occupancy (left to right) of the 20 Markov models with the highest posterior. All values are standardized by experimental value and uncertainty. Shaded areas indicate values for wild type, mutant E238Q, and mutant E485Q. Each panel shows the values of Markov model either with (circles) or without (diamonds) any direct allosteric interaction between the nucleotide binding sites and including mutation affecting affinities (gray) and additionally having different effect strength for the two different mutation (black).
